# Transcriptome Analysis of Triple-Negative HCC1937 and MDA-MB-231 Breast Cancer Cells Treated with *Kalanchoe pinnata* Revealed the Regulation of Migration and Invasion via the Downregulation of the Genes JAK2, ROCK1 and ROCK2

**DOI:** 10.1021/acsomega.5c05895

**Published:** 2025-07-09

**Authors:** Carlos Alvizo-Rodríguez, Alan Carrasco-Carballo, Uriel López-Vázquez, Georgina Hernández-Montes, Marta E. Hernández-Caballero

**Affiliations:** † Facultad de Medicina, Biomedicina, 3972 Benemérita Universidad Autónoma de Puebla. 13 sur 2702 Col. Volcanes, C.P., Puebla 72410, Mexico; ‡ Laboratorio de Elucidación y Síntesis en Química Orgánica, Instituto de Ciencias, Benemérita Universidad Autónoma de Puebla, Puebla 72410, Mexico; § Secretaría de Ciencia, Humanidades, Tecnología e Innovación (SECIHTI), Av. Insurgentes Sur 1582, Col. Crédito Constructor, Demarcación Territorial Benito Juárez. CP, Ciudad de Mexico 03940, Mexico; ∥ Coordinación de la Investigación Científica, UNAM - Red de Apoyo a la Investigación. Vasco de Quiroga 15, Sección XVI, Tlalpan C.P., Ciudad de Mexico 14000, Mexico

## Abstract

Triple-negative breast cancer (TNBC) is a type of breast cancer with high mortality due to aggressive tumor behavior and limited treatment options. Natural products have been studied for their potential as alternatives to combine with cancer drugs to improve treatment efficacy. Plant phytoconstituents can regulate cellular processes such as proliferation, differentiation, invasion, angiogenesis, and migration. *Kalanchoe pinnata* has been used in traditional medicine; it contains flavonoids, phenols, alkaloids, glycosides, bufadienolides, and tannins, among others. In the present investigation, we evaluated the effects of aqueous extract and the underlying molecular mechanisms in two breast cancer cell lines, HCC1937 and MDA-MB-231, with a focus on cellular migration. The cytotoxic activity results revealed that HCC1937 cells are more sensitive to aqueous extracts than are MDA-MB-231 cells. Compared with that of 48 h-treated MDA-MB-231 cells, cell migration was affected in HCC1937 cells after 24 h of treatment. The extract had greater anti-invasion effects on HCC1937 cells (37.7%, *p* < 0.01) than on MDA-MB-231 cells (47%, *p* < 0.05). An RNA-seq assay revealed 1850 differentially expressed genes (DEGs) in HCC1937 cells versus 1534 in MDA-MB-231 cells. WebGestalt analysis revealed the downregulation of genes involved in cell–cell adhesion, ruffle organization, and cell–substrate junctions, with more downregulated genes in HCC1937 cells. The downregulated genes analyzed with KOBAS-i were associated with MAPK signaling, focal adhesion, PI3K-Akt signaling, regulation of the actin cytoskeleton, and the ECM-receptor interaction pathway. In addition, IPA network analysis revealed that the RHO GTPase cycle, RHOA signaling, JAK/STAT signaling, actin cytoskeleton signaling and Integrin signaling pathways were inhibited. The data from the docking study indicated the binding potential of quercetin-3-O-arabinoside, kaempferol, quercetin, and apigenin with the JAK2, ROCK1, ROCK2, and PIK3CA proteins. Thus, our results demonstrate that *K. pinnata* inhibits the proliferation and invasion of TNBC cells by targeting genes involved in these important processes; thus, *K. pinnata* has the potential to be used in combination with chemotherapeutic drugs for cancer treatment.

## Graphical abstract

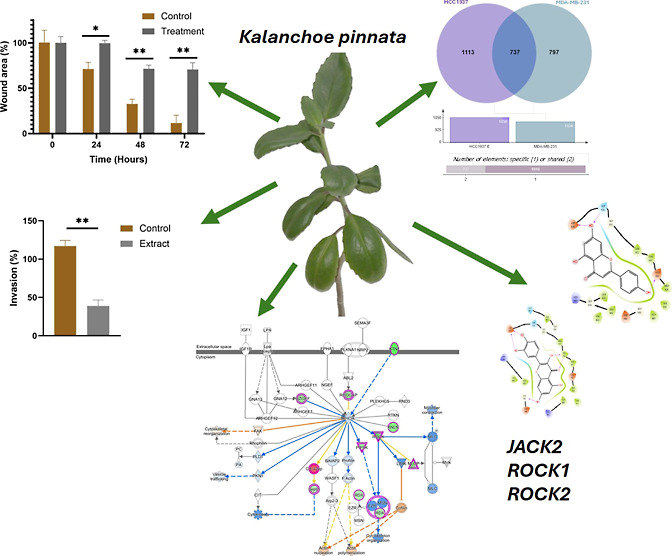

## Introduction

*Kalanchoe pinnata* (Lam) Pers (syn. Bryophyllum pinnata Lam.) belonging to the Crassulaceae family is a native plant from Madagascar that is distributed worldwide in tropical regions. Like other plants, *K. pinnata* has been used in traditional medicine in many countries to treat several afflictions, such as wounds, burns, rheumatism, and ulcers, as an antimicrobial, antiviral, antileishmanial, anthelmintic, anti-inflammatory or antitumor agent and as an antipyretic or muscle relaxant, among others.^1–3^ The metabolites present in *K. pinnata* include diverse flavonoids, phenols, alkaloids, triterpenes, glycosides, bufadienolides, tannins, amino acids, and microelements, and the findings of some researchers point to its effective antioxidant potential as one of its main metabolite properties.^4^

TNBC is one of the main types of breast cancer and represents a challenge because of its high mortality rate as a result of aggressive tumor behavior. TNBC is characterized by the absence of estrogen receptor (ER), progesterone receptor (PR), and HER2 expression and represents approximately 20% of all breast cancer types.^5^ Cancer treatment may include radiation, immunotherapy, and chemotherapy; some limitations to chemotherapy are solubility, instability, nonspecific distribution, systemic toxicity, and recurrence or relapse by acquired drug resistance, mostly in TNBC.^6^ In addition, chemotherapeutic drugs have side effects, so natural supplements have been studied for their potential as alternatives combined with cancer drugs to improve treatment efficacy.^7^ By targeting the expression of genes involved in the PI3K/Akt/mTOR, Nrf2/Keap1, MEK/ERK, Wnt/β or EGFR pathways, natural products can regulate various cellular processes that are crucial in cancer development, such as proliferation, differentiation, invasion, angiogenesis, and migration. It also includes the ability to act as an epigenetic regulator, and epigenetic alterations are the other key mechanism leading to cancer development.^8^ Therefore, there is a constant search for novel compounds with reduced toxicity from natural products.

Researchers have reported beneficial in vitro biological effects of treating breast cancer cell lines with extracts of *K. pinnata*. These studies used ethanolic extracts from traditional medicines. However, people commonly eat roasted leaves or prepare juice or leaf infusions, which means that they use something similar to an aqueous extract.^9^ However, no studies have analyzed the use of aqueous extracts, and only hydroethanolic extract studies have been reported.

Whole-transcriptome sequencing or RNA sequencing (RNA-seq) is a high-throughput technology used to quantify the expression of tens of thousands of RNA molecules and is a valuable tool for understanding the cellular processes in normal or nontreated cells vs altered or treated cells. High-throughput mRNA sequencing has accelerated the exploration of gene expression levels in diseases, especially cancer.

In this study, we analyzed RNA sequencing (RNA-seq) data from MDA-MB-231 and HCC1937 breast cancer cells treated with *K. pinnata* aqueous extract. We then assessed the DEGs to understand the molecular mechanisms involved in the cellular migration process. Our transcriptome analysis presents the first transcriptomic analysis of *K. pinnata* aqueous extract in breast cancer cells. Two signaling pathways relevant to metastatic cell behavior were identified, and the relationships of key genes with phytochemicals in the aqueous extract were correlated.

## Results

### Cytotoxic Effect of *K. pinnata* on HCC1937 and MDA-MB-231 Cells

As previously reported by our group, the total phenolic content of the *K. pinnata* aqueous extract used in this study was 357.34 ± 20.64 mg EGA/kg of plant, and the total flavonoid content was 408.90 ± 30.37 mg EQ/kg of plant. The CC_50_ value 72 h after treatment with the extract was 66 μg/mL in HCC1937 cells, as previously reported.^10^ The CC50 value 72 h after treatment with the extract was 140 μg/mL in MDA-MB-231 cells (Supporting Information Figure 1). The aqueous extract exhibited a dose-dependent cytotoxic effect, which was greater in HCC1937 cells than in MDA-MB-231 cells.

### Effect of Aqueous Extract of *K. pinnata* on Cell Migration

HCC1937 cells were exposed to 66 μg/mL aqueous extract, and MDA-MB-231 cells were exposed to 140 μg/mL aqueous extract for 72 h to assess the effect on cell migration via a wound healing assay. Compared with the untreated control, the *K. pinnata* extract had the greatest effect on HCC1937 cells, and significant differences were observed after 24 h of treatment when wound closure was retarded. The treated MDA-MB-231 cells did not achieve complete wound closure at the end of treatment, but statistical significance was achieved at 72 h of treatment (Figure 1).

**Figure 1:**
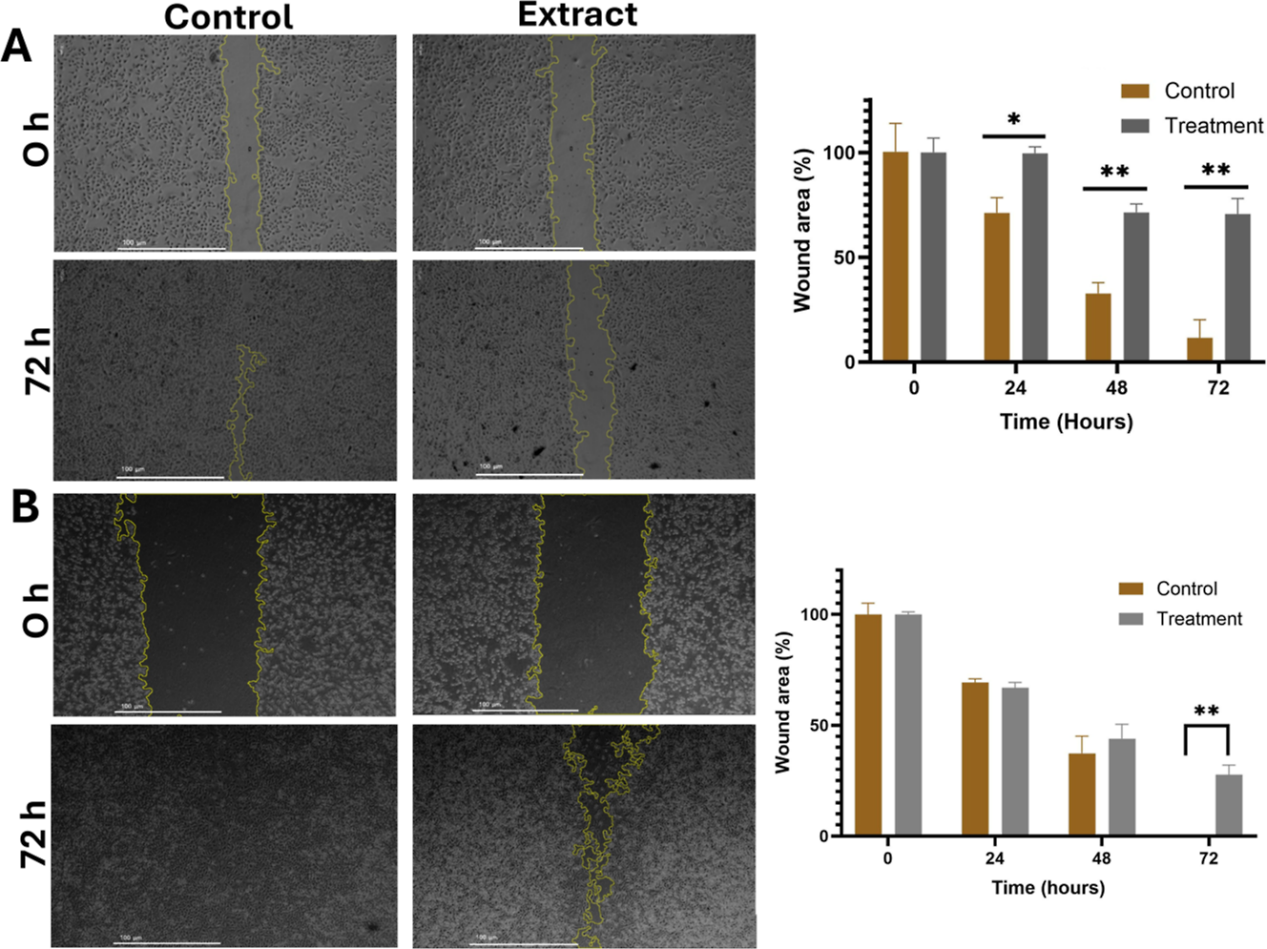
Effect of the aqueous extract of *K. pinnata* on cell migration after 72 h of treatment. Microscopy images of wound healing assays in control and treated HCC1937 (A) and MDA-MB-231 (B) cells. Images were taken at 0, 24, 48, and 72 h after scratch formation (scale bar = 100 μm). Percentage of wound closure in HCC1937 (C) and MDA-MB-231 (D) cells. The statistical significance is represented by asterisks (*p < 0.05; **p < 0.01) compared with the control without extract.

#### Effect of Aqueous Extract of *K. pinnata* on cell invasion

Since we observed that aqueous extracts inhibited cell migration, we verified the effects of *K. pinnata* on invasion capacity, and this assay confirmed that aqueous extracts of *K. pinnata* inhibited cell invasion after 48 h of treatment. A lower number of cancer cells migrated from the inside surface to the outside surface after treatment with the aqueous extract than after treatment with the negative control. Approximately 1 × 10^3^ HCC1937-untreated cells/mL migrated to the lower chamber, but with the aqueous extract, the number was reduced to only 377 cells/mL (37.7%, *p* < 0.01) (Figure 2), whereas with the untreated MDA-MB-231 cells, 7 × 10^3^ cells/mL migrated to the lower chamber, and with the aqueous extract, approximately 3 × 10^3^ cells/mL migrated (47%, *p* < 0.05) (Figure 2). A greater effect was observed in HCC1937 cells than in MDA-MB-231 cells. The results showed that aqueous extracts have the potential to suppress the invasive ability of cells.

**Figure 2:**
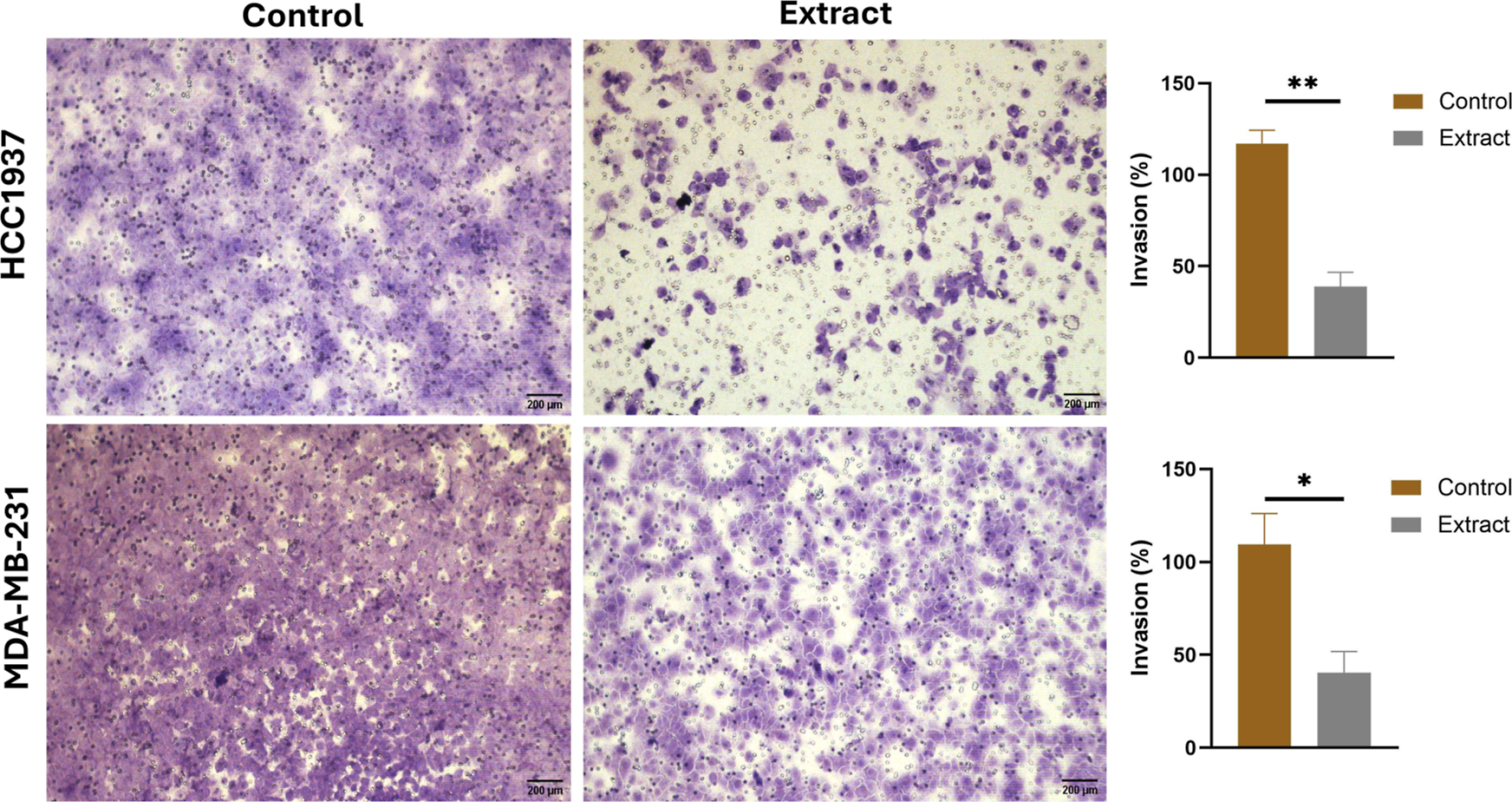
Transwell assay for cell invasion after treatment with the aqueous extract of *K. pinnata*. Microscopy images of HCC1937 and MDA-MB-231 cells. Images were taken 20 h after treatment (scale bar = 200 μm). The statistical significance is represented by asterisks (*p < 0.05; **p < 0.01) compared with the control without extract.

### Functional Enrichment Analysis

We performed RNA transcriptome sequencing in the MDA-MB-231 and HCC1937 cell lines after 72 h of treatment with the *K. pinnata* aqueous extract to characterize transcriptional changes upon treatment. Following quality control, a minimum of 94% clean data were obtained for each sample. The three biological replicates were significantly correlated, with correlation coefficients exceeding 0.9. Furthermore, the PCA results revealed that the 12 samples were grouped into four categories, with similar biological replicates clustered on the basis of cell type and treatment. The sequencing data were of high quality and consistent among replicates, making them suitable for further analysis. Differential gene expression was defined as a |log2 FC| ≥ 2.0. The expression profile of both cell lines was significantly altered as a result of treatment. Venn analysis revealed that HCC1937 cells presented more DEGs, with 1850 genes in MDA-MB-231 cells and 1534 genes in cells treated with the aqueous extract (Figure 3).

**Figure 3:**
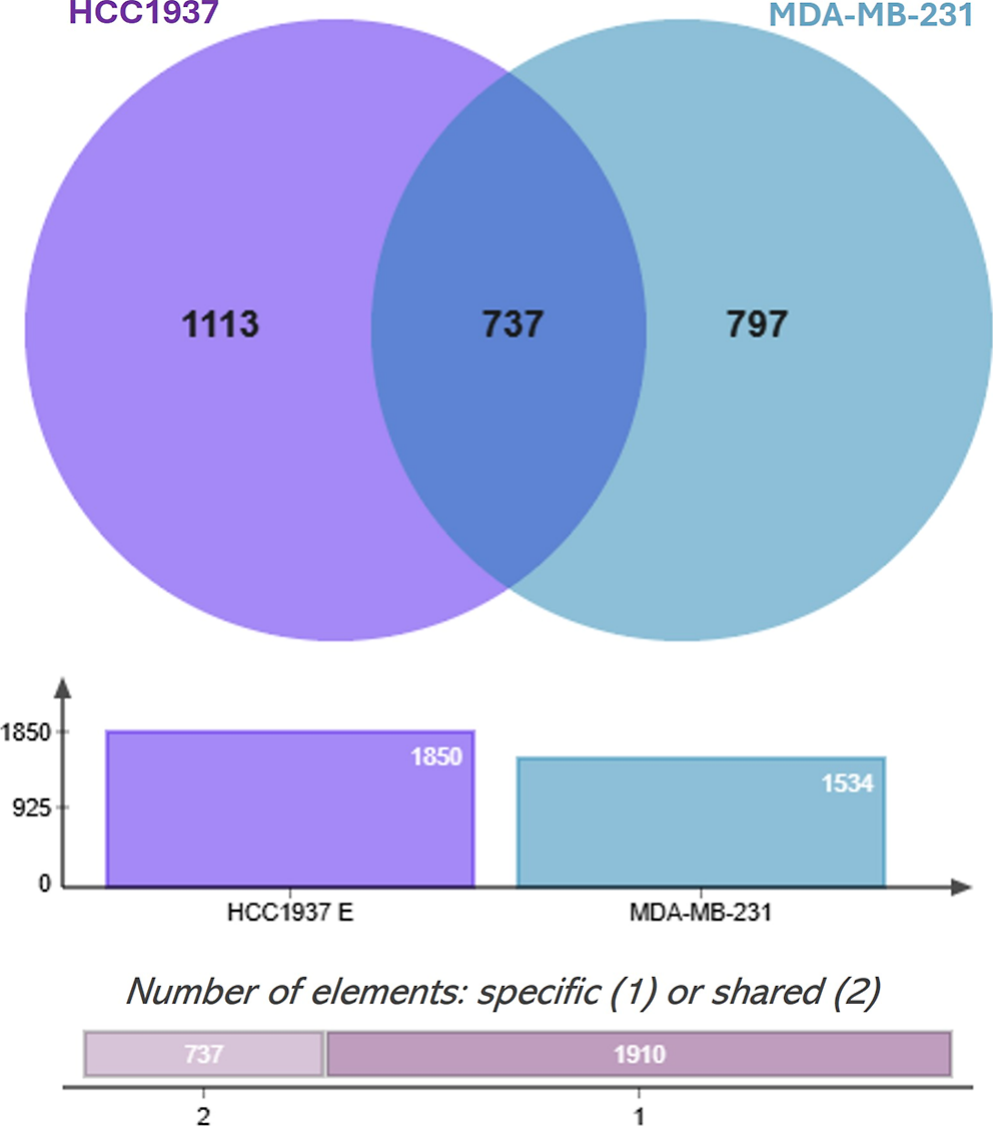
Shared genes between HCC1937 and MDA-MB-231 cells after treatment with aqueous extracts of *K. pinnata*. HCC1937 and MDA-MB-231 cells were treated with aqueous extracts. There were 737 shared genes when the cells were treated with the aqueous extract.

### ORA Assay

To determine if the aqueous extract generates evident changes in gene expression, we analyzed its effect on breast cancer cells. Based on the response of cells to extract treatment, ORA analysis was performed using WebGestalt with the parameters *Homo sapiens* reference set genome, and significance level TOP obtaining 1848 candidate genes for HCC1937 cells classified into categories: biological process (BP), cellular component (CC), and molecular function (MF). In the top significance categories, we are interested in genes participating in the migration process. Figure 4 shows that the HCC1937 cells treated with the extract showed 210 related to cell population proliferation in the category BP. In the category of CC, 274 genes were related to cell projection and 53 to the extracellular matrix. In the MDA-MB-231 cell line, 1541 were classified in enrichment categories, and there were more differences in categories of interest, cells treated with the aqueous extract showed 158 genes involved in cell population proliferation in category BP, 216 in cell projection and 44 in extracellular matrix in the category CC (Figure 5). The gene sets were enriched in cadherin superfamily members of transmembrane proteins involved in cell adhesion, protocadherins, cadherin-related family members, and claudins. The enrichment of genes in processes related to cell cycle, chromosome segregation and organization for both cell lines are shown in Supporting Information Table 1. Genes involved in the proliferation process included cyclins, cyclin-dependent kinases, centrosomal proteins, structural maintenance of chromosome proteins, and diverse regulators.

**Figure 4:**
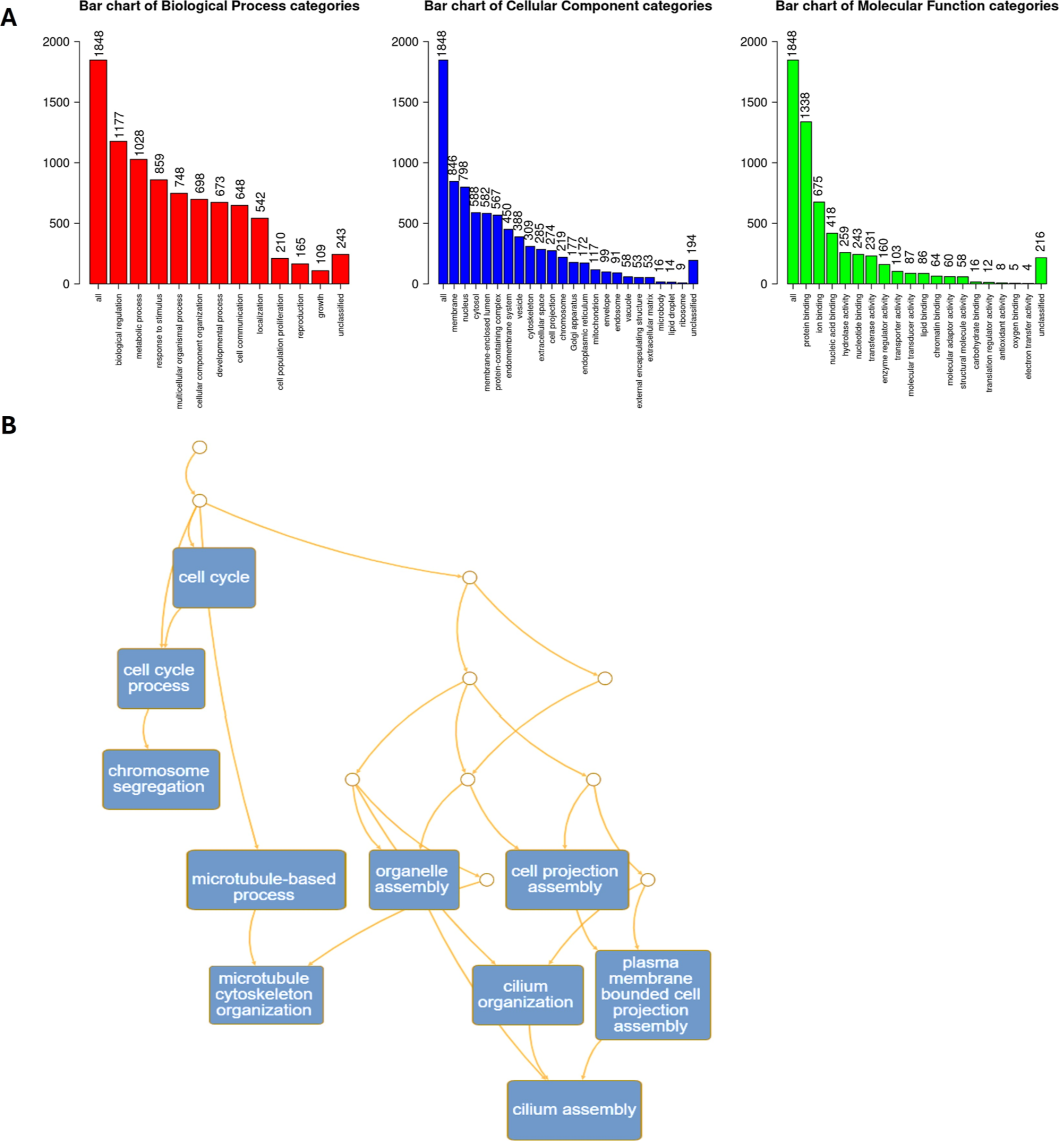
GO Slim summary of overrepresentation analysis (ORA) of HCC1937 cells after 72 h of aqueous extract of *K. pinnata* treatment (A) Biological process, cellular component, and molecular function categories are represented by red, blue, and green bars, respectively. The height represents the enriched number of genes in each category (B) Network structure for functional categories via a directed acyclic graph (DAG).

**Figure 5:**
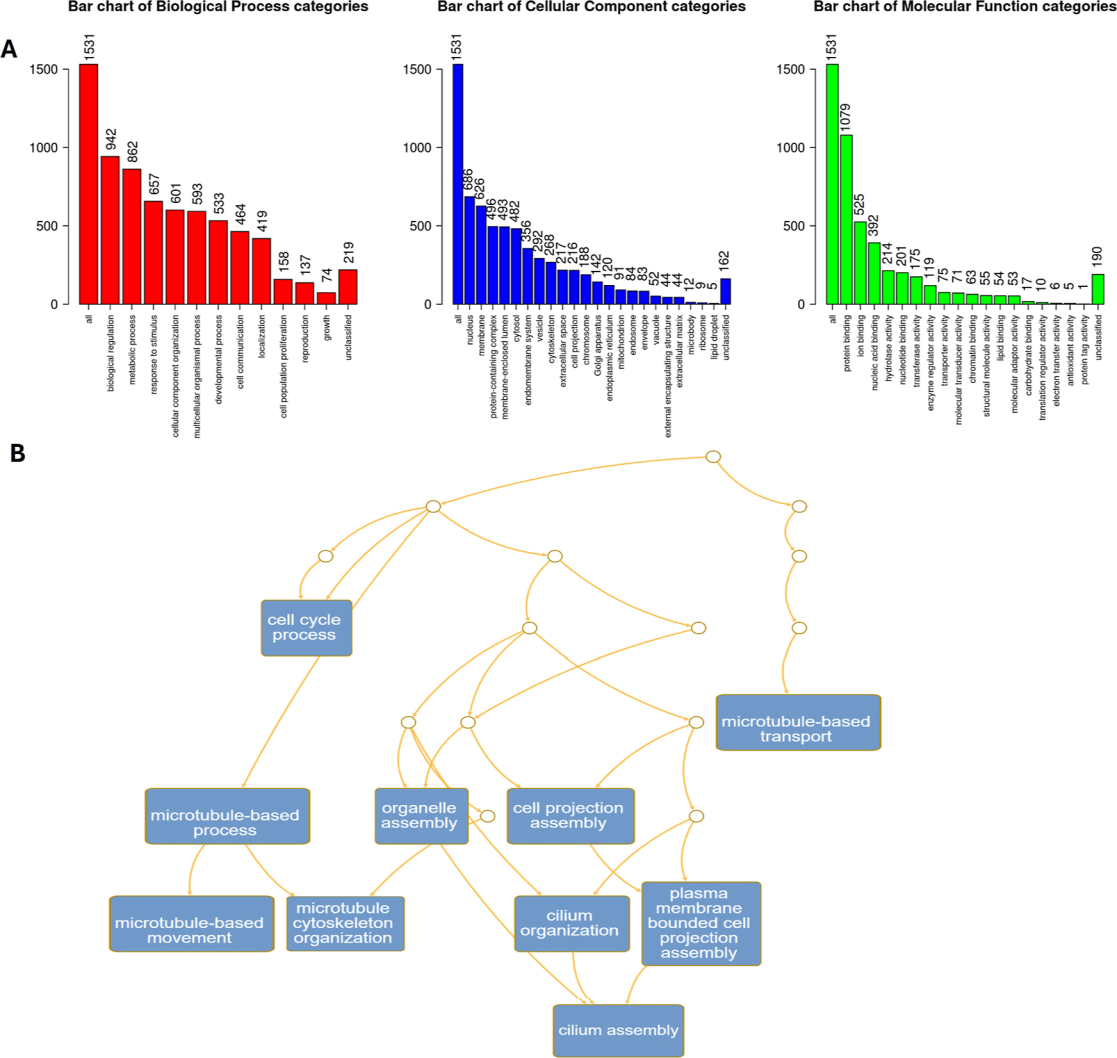
GO analysis of MDA-MB-231 cells after 72 h of aqueous extract of *K. pinnata* treatment. GO analysis (A) Biological process, cellular component, and molecular function categories are represented by red, blue, and green bars, respectively. The height represents the enriched number of genes in each category. (B) Network structure for functional categories via a directed acyclic graph (DAG).

### GSEA Assay

GSEA was performed to obtain insights into the biological roles of the DEGs focused on cell migration. The genes whose expression was downregulated in HCC1937 cells treated with the *K. pinnata* aqueous extract were significantly enriched in genes related to cell–cell adhesion via plasma–membrane adhesion molecules; ruffle organization; and cell–substrate junctions, including the protocadherin genes PCDHGA5, PCDHGA12, PCDHGB3, PCDHGB4, and PCDHGC4; and cadherin-related family members CDHR2, CDHR5, cadherin CDH26, ADAM proteases and claudin (Table 1). Other significantly enriched processes included mRNA processing, kinetochore, and spindle organization or the inflammatory response (Supporting Information Table 2). The upregulated genes were significantly enriched in receptor and transporter complex and ncRNA processing genes, including METTL1, EIF6, MRT4, PLD6, and TRMT61A, but there was no significant enrichment related to cellular adhesion (Supporting Information Table 2).

**Table 1:** GSEA Analysis of HCC1937 Breast Cancer Cells Treated with *K. pinnata* Aqueous Extract

biological process GO:0098,742 cell–cell adhesion via plasma-membrane adhesion molecules^a^		
gene	gene name	score
PCDHGA12	protocadherin gamma subfamily A, 12	–5.5607
ROBO1	roundabout guidance receptor 1	–5.5354
PCDHB4	protocadherin beta 4	–5.4195
PCDHGC4	protocadherin gamma subfamily C, 4	–5.3741
PCDHGB3	protocadherin gamma subfamily B, 3	–4.7372
CLDN18	claudin 18	–4.659
EMB	embigin	–4.5184
CDHR2	cadherin related family member 2	–4.1646
PCDHGB4	protocadherin gamma subfamily B, 4	–4.0687
PCDHGA6	protocadherin gamma subfamily A, 6	–4.0323
CDHR4	cadherin related family member 4	–3.9204
TENM2	teneurin transmembrane protein 2	–3.7605
LRRC4	leucine rich repeat containing 4	–3.6594
PCDHGA5	protocadherin gamma subfamily A, 5	–3.5792
CDHR5	cadherin related family member 5	–3.3612
cellular component GO:0005912 adherens junction^a^		
CDH26	cadherin 26	–2.485
RDX	radixin	–2.4609
LDB3	LIM domain binding 3	–2.4095
DLG1	discs large MAGUK scaffold protein 1	–2.3456
ADAM10	ADAM metallopeptidase domain 10	–2.3396
CXADR	CXADR Ig-like cell adhesion molecule	–2.3045
SSX2IP	SSX family member 2 interacting protein	–2.2783
FER	FER tyrosine kinase	–2.1966
LIN7C	Lin-7 homologue C, crumbs cell polarity complex component	–2.1899
VEZT	vezatin, adherens junctions transmembrane protein	–2.0788
FRS2	fibroblast growth factor receptor substrate 2	–2.0023
cellular component GO:0030,055 cell-substrate junction^a^		
DST	dystonin	–3.1675
SYNPO2	synaptopodin 2	–3.1073
CAV3	caveolin 3	–3.0502
SYNE2	spectrin repeat containing nuclear envelope protein 2	–3.0487
ERBIN	Erbb2 interacting protein	–3.0212
ITGA2B	integrin subunit alpha 2b	–2.8091
NHS	NHS actin remodeling regulator	–2.7629
PPP1R12A	protein phosphatase 1 regulatory subunit 12A	–2.7011
ITGB8	integrin subunit beta 8	–2.5962
ADAM9	ADAM metallopeptidase domain 9	–2.5885
ITGAV	integrin subunit alpha V	–2.5289
NCKAP1	NCK associated protein 1	–2.4871
RDX	radixin	–2.4609
USP33	ubiquitin specific peptidase 33	–2.4564
RHOU	Ras homologue family member U	–2.4157
JAK2	janus kinase 2	–2.4012
CD46	CD46 molecule	–2.3578
HSP90B1	heat shock protein 90 beta family member 1	–2.3501
ADAM10	ADAM metallopeptidase domain 10	–2.3396
KIF23	kinesin family member 23	–2.3276
VIM	vimentin	–2.3152
ALCAM	activated leukocyte cell adhesion molecule	–2.3006
ADGRB1	adhesion G protein-coupled receptor B1	–2.2854
TWF1	twinfilin actin binding protein 1	–2.2305
LMO7	LIM domain 7	–2.2171
YES1	YES proto-oncogene 1, Src family tyrosine kinase	–2.1894
RND3	Rho family gtpase 3	–2.1849
IQGAP1	IQ motif containing gtpase activating protein 1	–2.0242

a Top 10 FDR ≤ 0.05.

GSEA of MDA-MB-231 cells treated with the *K. pinnata* aqueous extract revealed that downregulated genes related to cell–cell adhesion via plasma–membrane adhesion molecules, such as protocadherins PCDHGC4, PCDHGB3, PCDHGB4, cadherin-related family members CDHR2, CDHR5, claudin CLDN18, and CLDN20 and AMIGO3, were enriched, similar to those in HCC1937 cells (Table 2). Additionally, the cells were significantly enriched in kinetochore organization, chromosome organization, or GTPase activity (Supporting Information Table 3). The upregulated genes were enriched in nuclear processes such as the rRNA metabolic process, nuclear transport, intermediate filament cytoskeleton, or respirasome (Supporting Information Table 3).

**Table 2:** GSEA Analysis of MDA-MB-231 Breast Cancer Cells Treated with *K. pinnata* Aqueous Extract

biological process GO:0098,742 Cell–cell adhesion via plasma-membrane adhesion molecules^a^		
gene	gene name	score
AMIGO3	adhesion molecule with Ig like domain 3	–7.7735
PCDHGA7	protocadherin gamma subfamily A, 7	–5.4592
PCDHGB3	protocadherin gamma subfamily B, 3	–5.3436
CDHR2	cadherin related family member 2	–5.1399
PCDHGA9	protocadherin gamma subfamily A, 9	–5.0388
PCDHGA8	protocadherin gamma subfamily A, 8	–4.9285
PCDHGC4	protocadherin gamma subfamily C, 4	–4.8778
CLDN18	claudin 18	–4.8316
PCDHA9	protocadherin alpha 9	–4.3923
PCDHA10	protocadherin alpha 10	–4.2527
PCDH19	protocadherin 19	–4.1041
CNTN2	contactin 2	–3.9753
PCDHA5	protocadherin alpha 5	–3.6821
SLITRK2	SLIT and NTRK like family member 2	–3.6561
PCDHGB4	protocadherin gamma subfamily B, 4	–3.6041
CLDN20	claudin 20	–3.5406
CDHR5	cadherin related family member 5	–3.5251

a Top 10 FDR ≤ 0.05.

### KEGG Pathways

KEGG analysis via KOBAS-i revealed that in both the HCC1937 and MDA-MB-231 cell lines, the downregulated genes were associated with pathways such as MAPK signaling, focal adhesion, PI3K-Akt signaling, or regulation of the actin cytoskeleton. In addition, MDA-MB-231 cells were enriched in the ECM–receptor interaction pathway, which was not observed in HCC1937 cells. The pathways associated with the upregulated genes were the same as those associated with the downregulated genes (Supporting Information Tables 4 and 5). IPA using the canonical pathway module with attention to cytoskeleton organization and proliferation revealed the inhibition of signaling pathways such as the RHO GTPase cycle [log­(p value) 11.9; ratio 0.0978; *z* score −6.03]; RHOA signaling [log­(*p* value) 4.94; ratio 0.114; *z* score −0.832]; JAK/STAT signaling [log­(*p* value) 2.6; ratio 0.0964; *z* score −1.89]; actin cytoskeleton signaling [log­(*p* value) 1.99; ratio 0.0576; *z* score −1.897]; Integrin signaling [log­(*p* value) 1.42; ratio 0.526; *z* score −3] in HCC1937 cells; RHO GTPase cycle [log­(*p* value) 6.18; ratio 0.0578; *z* score −4.707]; RHOA signaling [log­(*p* value) 5.37; ratio 0.976; *z* score −0.302]; JAK/STAT signaling [log­(*p* value) 2.29; ratio 0.0723; *z* score −1.342]. These findings indicate that *K. pinnata* aqueous extract affects cellular migration and proliferation (Figures 6 and 7).

**Figure 6:**
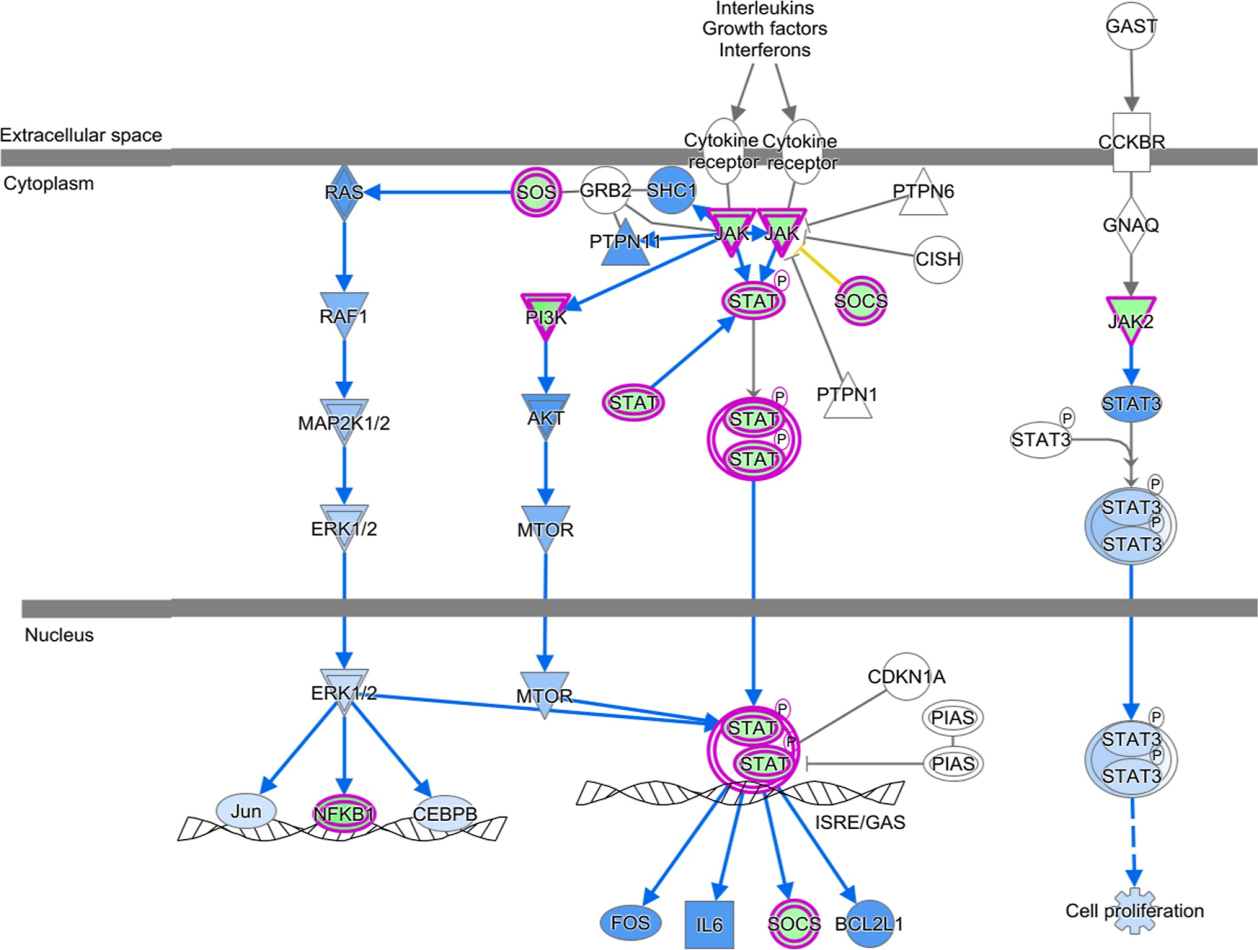
JAK/STAT signaling pathway. JAK/STAT signaling in HCC1937 cells treated with aqueous extracts of *K. pinnata* and MDA-MB-231 cells. Genes downregulated (green) Molecules predicted to be downregulated (blue), molecules predicted to be inhibited (blue line), and indirect relationships (dashed lines). The intensity of the color indicates the magnitude of gene expression regulation. The gloss glow indicates activity when the measurement is the opposite, on the basis of the IPA knowledgebase.

**Figure 7:**
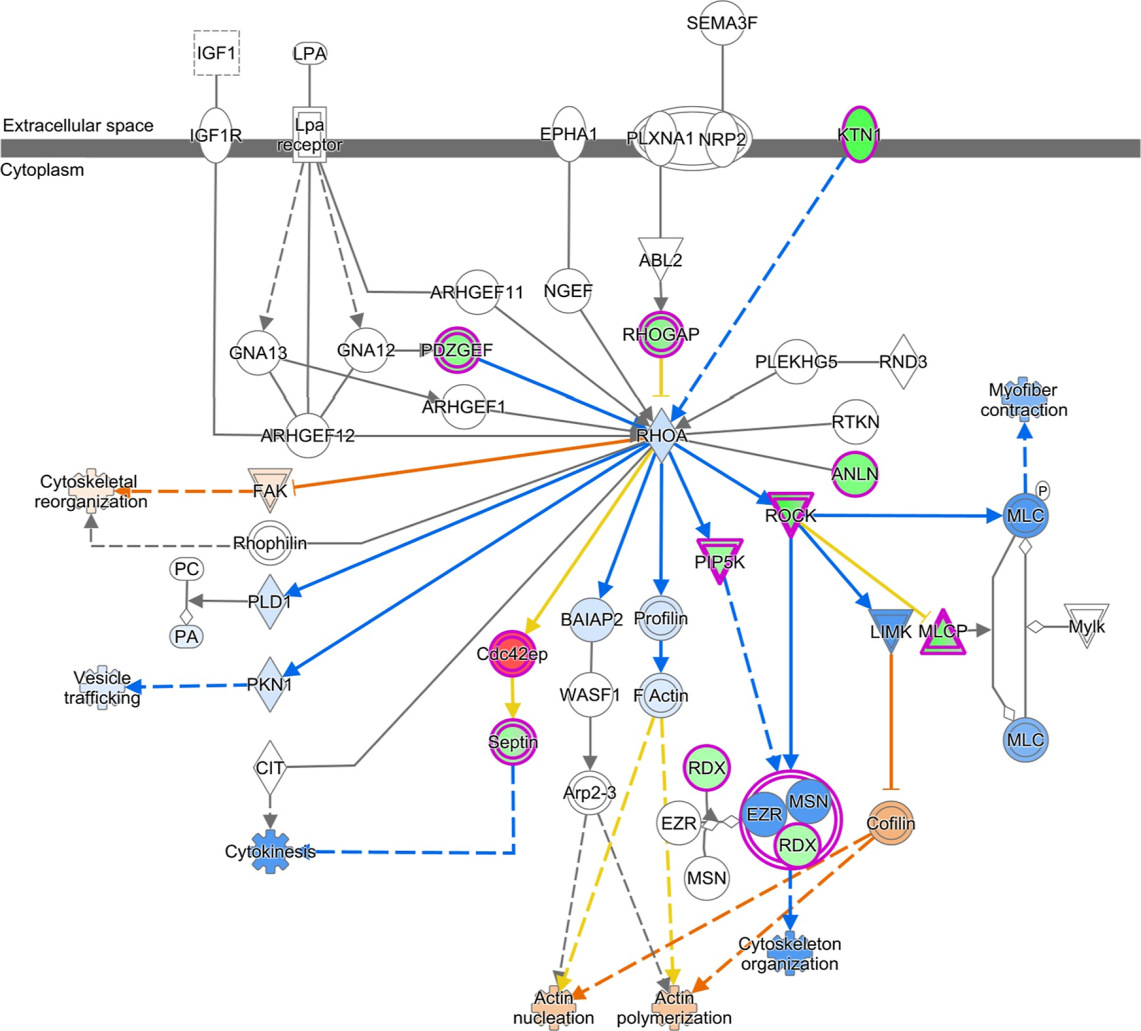
RHOA signaling pathway RHOA signaling in HCC1937 cells treated with aqueous extracts of *K. pinnata* and MDA-MB-231 cells. Genes that are overexpressed (red), downregulated (green), molecules that are predicted to be activated (orange line), molecules that are predicted to be downregulated (blue), molecules that are predicted to be inhibited (blue line), and indirect relationships (dashed lines). The intensity of the color indicates the magnitude of gene expression regulation. The gloss glow indicates activity when the measurement is the opposite, on the basis of the IPA knowledgebase.

#### Molecular Docking

The analysis was performed on the proteins resulting from the activation pathways involved, which are presented in Table 3, for the polar secondary metabolites reported for *K. pinnata*.^10^

**Table 3:** Docking Score of Secondary Metabolites of *Kalanchoe pinnata*

metabolite	JAK2	ROCK1	ROCK2	PIK3CA
quercetin-3-O-arabinoside	–8.798	–7.969	–6.895	–9.510
kaempferol	–7.086	–9.093	–7.511	NI
quercetin	–8.365	–8.440	–6.567	–6.731
apigenin	–6.911	–8.640	–7.463	–8.846
caffeic acid	–5.541	–5.520	–4.568	–7.165
3,4-dihydroxybenzoic acid	–5.479	–5.001	–4.909	–6.246
astragalin	–5.819	–6.757	–6.132	–7.523
3,8-dimethylherbacetin	–6.771	–7.990	–7.561	–7.546
luteolin	–8.043	–8.345	–6.225	–9.364
rutin	–6.968	–6.862	–5.530	–9.317
kaempferol 3-O-(alpha-l-rhamnopyranosyl(1→2)-beta-d-galactopyranosyl)-7-O-alpha-l-rhamnopyranoside	–6.747	–6.439	–5.845	–8.209
quercetin 3-O-[alpha-l-rhamnopyranosyl(1→2)-beta-d-galactopyranosyl]-7-O-alpha-l-rhamnopyranoside	–5.211	–5.435	–6.107	–6.893
myricitrin	–5.262	–7.794	–4.956	–7.261
patuletin	–5.047	–3.369	–4.335	–6.636
bryophyllin B	–5.001	–2.974	–4.632	–5.087
reference of protein^a^	–5.344	–7.962	–6.501	–6.986

a Reference, JAK2: Fedretanib; ROCK1: Y-27632 dihydrochloride; ROCK2: Belumusudil; PIK3CA: Alpelisib.

The interactions are mainly highlighted by the formation of hydrogen bonds by the phenolic hydroxyls of the flavonoids, as in the case of kaempferol, although to a lesser extent, the insertion of glucosides improves the coupling; however, many carbohydrates increase the volume, decreasing the interaction, as observed in the other flavonoids with carbohydrates. This phenomenon is independent of the type of protein in which one works, as shown in JAK2 (Figure 8a–d) by the formation of hydrogen bonds mainly toward ASN981, ASP393 and SER936, whereas for PIK3CA (e-g), it is associated with TYR836, ASP850 and LYS802.

**Figure 8:**
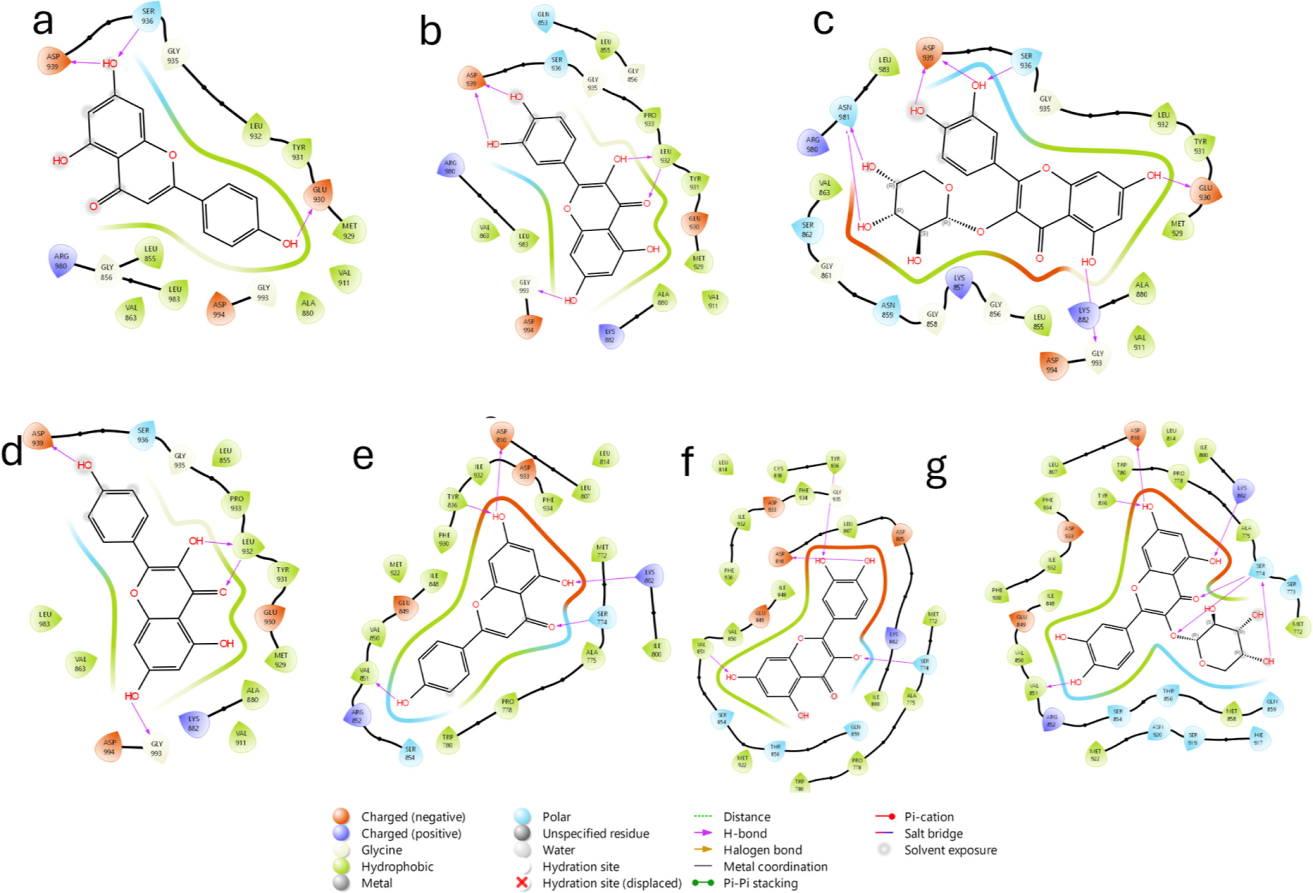
2D interaction of (a) apigenin in JAK2, (b) quercetin in JAK2, (c) quercetin-3-O-arabinoside in JAK2, (d) kaempferol in JAK2, (e) apigenin in PIK3CA, (f) quercetin in PIK3CA, and (g) quercetin-3-O-arabinoside in PIK3CA.

**Figure 9:**
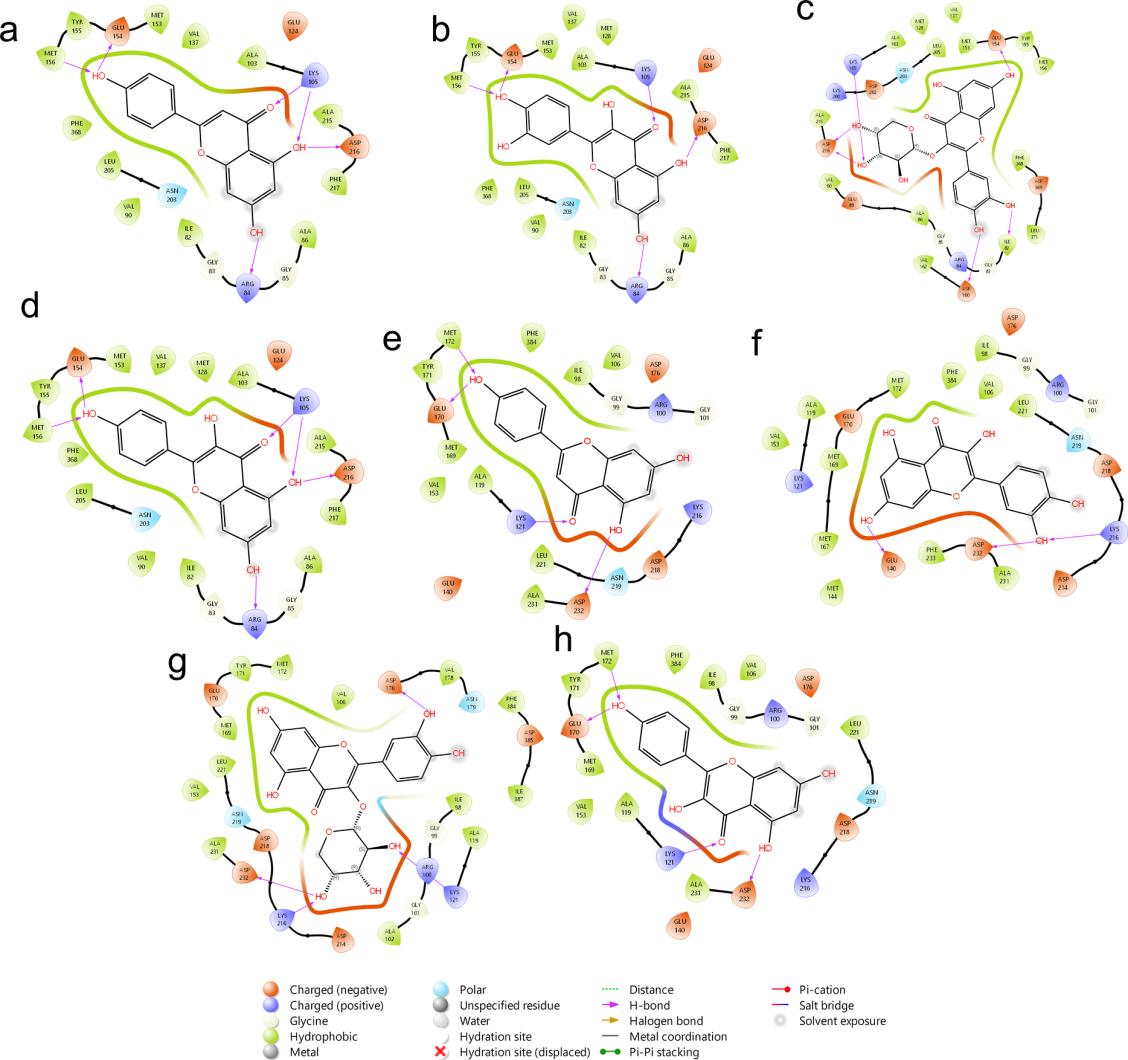
2D interaction of (a) apigenin in ROCK1, (b) quercetin in ROCK1, (c) quercetin-3-O-arabinoside in ROCK1, (d) kaempferol in ROCK1, (e) apigenin in ROCK2, (f) quercetin in ROCK2, (g) quercetin-3-O-arabinoside in ROCK2 and (h) kaempferol in ROCK2.

Similarly, for JAK2 and PIK3CA, polar interactions predominate on the phenolic hydroxyls. In addition to the carbonyl group of the flavonoid skeleton, for ROCK1, the interactions are centered toward MET153 and GLU154 on the part of the hydroxyls, whereas for the carbonyl, the interaction is with LYS105 (Figure 9). For ROCK2, it is similar, given the similarity between proteins, focused on MET172 and GLU170 for the hydroxyls, in addition to ASP232, they are conserved toward the carbonyl group, mainly toward LYS121.

## Discussion

Triple-negative breast cancer is a subtype of breast cancer well-known for its capacity to metastasize despite treatment, so the complexity of this pathology results in a poorer prognosis than the other subtypes.^5^ Diverse evidence from clinical trials offers more options involving chemotherapeutic, immunotherapeutic, and targeted therapies or treatment combinations.^11^ Traditional medicine is an important source of natural products with the potential to be included in breast cancer as a complementary treatment. In addition, more evidence has accumulated every day from ethnopharmacological investigations that analyze the bioactive compounds of medicinal plants. *K. pinnata* has a long history of use for treating burns, rheumatism, or as an antipyretic among other conditions, including cancer. Many researchers have reported beneficial in vitro biological effects by treating breast cancer cell lines with extracts of *K. pinnata*, and these studies were established on traditional medicine use. This study was based on an analysis of the changes in the transcriptomes of two cell lines, with a focus on genes involved in migration and related processes. We used a cell line without ER or PR with a BRCA1 mutation, HCC1937, and a cell line with normal BRCA1 without ER or PR, MDA-MB-231, to analyze the effects of the aqueous extract. People commonly eat roasted leaves or prepare juice or leaf infusions from *K. pinnata*.^9^ The effects of hydroethanolic extracts have been reported, but few studies have analyzed the use of only aqueous extracts. For example, Stefanowicz-Hajduk et al. reported high cytotoxic activity of ethanolic extracts on MCF-7 cells. Quercetin and kaempferol are the major phenolic compounds present in the ethanolic extract of *K. pinnata*, and their effects include damage to the mitochondrial membrane potential, the induction of reactive oxygen species, and apoptosis.^12,13^ Recently, Machinski et al.^4^ identified the active components in the aqueous extracts of three *Kalanchoe* species, including *K. pinnata*, and they reported quercetin and quercetin 3-O-α-1-arabinopyranosyl (1→2) α-l-rhamnopyranoside, which was also reported in an aqueous extract by dos Santos Nascimento et al.^14^ de Araújo et al. reported patuletin, eupafolin and kaempferol in addition to quercetin in aqueous extracts of *K. pinnata*.^15^ Muzitano et al. reported the presence of quercitrin.^16^ Data obtained from published studies on the use of *K. pinnata*, including our results, shed light on the functions that flavonoids of *K. pinnata* may have in cancer since they have been found to have cytotoxic and apoptotic activity. In the present study, we found that aqueous extract treatment had a cytotoxic effect in a concentration-dependent manner, and the greatest cytotoxicity was observed in the HCC1937 cells at 66 μg/mL versus 140 μg/mL in the MDA-MB-231 cells. As indicated by Machinski et al.,^4^ many factors can influence the CC_50_ obtained. The season of collection is also important, as demonstrated by Muzitano et al., since the greatest abundance of flavonoids is found in the leaves in summer.^16^ In our case, the cellular types studied presented differences in the BRCA1 gene, with HCC1937 cells exhibiting a mutation that can increase the sensitivity of the cells to *K. pinnata* phytochemicals.^10^ The effects of *K. pinnata* on cancer cell invasion have not been reported. However, considering its effect on cell migration and the importance of invasion to tumor progression, we explored the effect on cell invasion and demonstrated that *K. pinnata* extract could effectively inhibit the invasion capacity of HCC1937 and MDA-MB-231 cells, especially HCC1937 cells (37.7%, *p* < 0.01), with respect to MDA-MB-231 cells (47%, *p* < 0.05). These findings suggest that *K. pinnata* may help suppress TNBC metastasis. On the basis of these results and all studies previously published that support the role of *K. pinnata* in proliferation, migration, and apoptosis, we investigated the changes in the transcriptome of HCC1937 and MDA-MB-231 cells after treatment with the aqueous extract of *K. pinnata*.

Our research focused predominantly on identifying DEGs involved in cell migration and demonstrated that phytochemicals present in *K. pinnata* significantly impact migration, invasion, and proliferation by targeting key genes in both cell lines. We found that genes such as ARHGAP5, BIRC3, ITPR2, PPP1R12A, PPP1R12B, PLCE1, ROCK1, ROCK2, SOS1, SOS2, JAK2, HSPA6, HSP90B1, PKN2, and PIK3CA were subexpressed after treatment. These genes are involved in proteoglycans in cancer, focal adhesion, apoptosis, MAPK signaling, and/or PI3K-Akt signaling pathways. In the focal adhesion pathway, several genes are downregulated, such as those encoding extracellular matrix (ECM) components, including COL4A5 (collagen type IV alpha 5 chain), LAMA1 (laminin subunit alpha-1) or FN1 (fibronectin 1); the integral membrane proteins ITGA5 (integrin subunit alpha 5), ITGA9 (integrin subunit alpha 9), ITGA2B (integrin subunit alpha 2b), and ITGAV (integrin subunit alpha V); and the cytoplasmic components ARHGAP5 (Rho GTPase-activating protein 5), ROCK1 (Rho-associated coiled-coil-containing protein kinase 1), ROCK2 (Rho-associated coiled-coil-coil–coil-containing protein kinase 2), PPP1R12A (protein phosphatase 1 regulatory subunit 12A), PPP1R12B (protein phosphatase 1 regulatory subunit 12B), and PKN2 (protein kinase N2), resulting in the inhibition of cell migration and invasion and actin polymerization to filopodia and lamellipodia formation.

The possible antitumor effect of polar compounds such as Quercetin-3-O-arabinoside, Kaempferol, Quercetin, and Apigenin was investigated by molecular docking. The results of our docking indicate that JAK2, ROCK1, ROCK2, and PIK3CA had the highest inhibitory activity with these phytochemicals, emphasizing that the flavonoid nucleus is the one that generates the coupling on these pathways generating a possible alteration in them resulting in the deregulation of cell proliferation pathways, this also depends on the composition, these metabolites being the ones that in addition to presenting scores higher than reference and/or commercial inhibitors.

Compared with normal cells, tumor cells exhibit increased cell migration and invasion which are essential for metastasis; TNBC is notable for its potential early metastasis.^17^ Rho-associated coiled-coil kinases (ROCKs) are important for actin cytoskeleton assembly control and proliferation, adhesion, migration, and phagocytosis through the phosphorylation of abundant downstream targets, including actin-related proteins and intermediate filament proteins. The activity of ROCKs is regulated by Rho proteins such as RhoA.^18^ The expression of ROCKs is increased in metastatic breast cancer.^19^ Yang et al. reported that ROCK1 overexpression in vivo and in vitro promoted the metastasis of laryngeal squamous cell carcinoma via the JAK2/STAT3/ERK1/2 pathway.^20^ The JAK2/STAT3 signaling pathway is overly activated in tumors and participates in resistance to paclitaxel by upregulating antiapoptotic gene expression.^21^ Quinazolines, dihydroartemisinin or pantoprazole are considered to inhibit the JAK2/STAT3 pathway in combination with standard treatment. In breast cancer, C–C motif chemokine ligand 20 (CCL20) induces the phosphorylation of JAK2, so it has been targeted to inhibit JAK2 and consequently the JAK2/STAT3 pathway.^22^ JAK2 phosphorylates STAT3, which is relocated to the nucleus and acts as a transcription factor; thus, the downregulation of JAK2 by *K. pinnata* can also inhibit this pathway. This can affect diverse proteins, such as those involved in the organization of the cytoskeleton, as reported by Haciosmanoglu Aldogan et al.^23^ Wang et al. suggested that the expression of ROCK2 is tightly associated with STAT3 activation.^24^ ROCKs control stress fiber and focal adhesion formation, cell migration, and centrosome maintenance through the phosphorylation of diverse substrates, and their inhibition affects chromosome segregation and blocks cytokinesis.^25^ According to a study by Cahuzac et al., ROCK2, which participates in the G1/S phase transition, is a good target for reversing the resistance of prostate cancer cell lines to olaparib.^26^ Other plants, such as Scutellaria barbata D. Don, affect RhoA/ROCK1 signaling in MDA-B-231 and BT-20 TNBC cells.^27^ The altered expression of ROCK1 stimulates the migration of cancer cells and is suggested to promote cell proliferation.^28^ PIK3CA encodes the catalytic subunit p110α of the PI3K protein (a key enzyme in the PI3K/AKT/mTOR pathway), which is frequently mutated in cancer; these mutations activate kinase activity and may lead to sustained activation of downstream Akt.^29^ PIK3CA not only promotes sustained proliferative signals but also may influence aggressive behavior and invasiveness.^30^ TNBC is considered to have a high prevalence of PI3K/AKT pathway activation.^31^ In this work, we did not observe the overexpression of the transcription factors Akt or FOXO, two important actors in that pathway, which may be the result of the absence of PIK3CA. To date, there are no reports on the regulatory effects of *K. pinnata* on ROCK1/2, JAK2 or PIK3CA. On the basis of these protein functions and the ability of cancer cells to migrate by altering their cytoskeleton and adhesion properties or interacting with EMC, in addition to inhibiting apoptosis, we suggest that the phytochemicals present in *K. pinnata* may function to decrease the activation of migration, growth and survival signal transduction pathways, including the MAPK, PI3K/AKT, and JAK2/STAT3 pathways, which are required for cancer cell maintenance, mainly in HCC937 cells (Figures 10 and 11).

**Figure 10:**
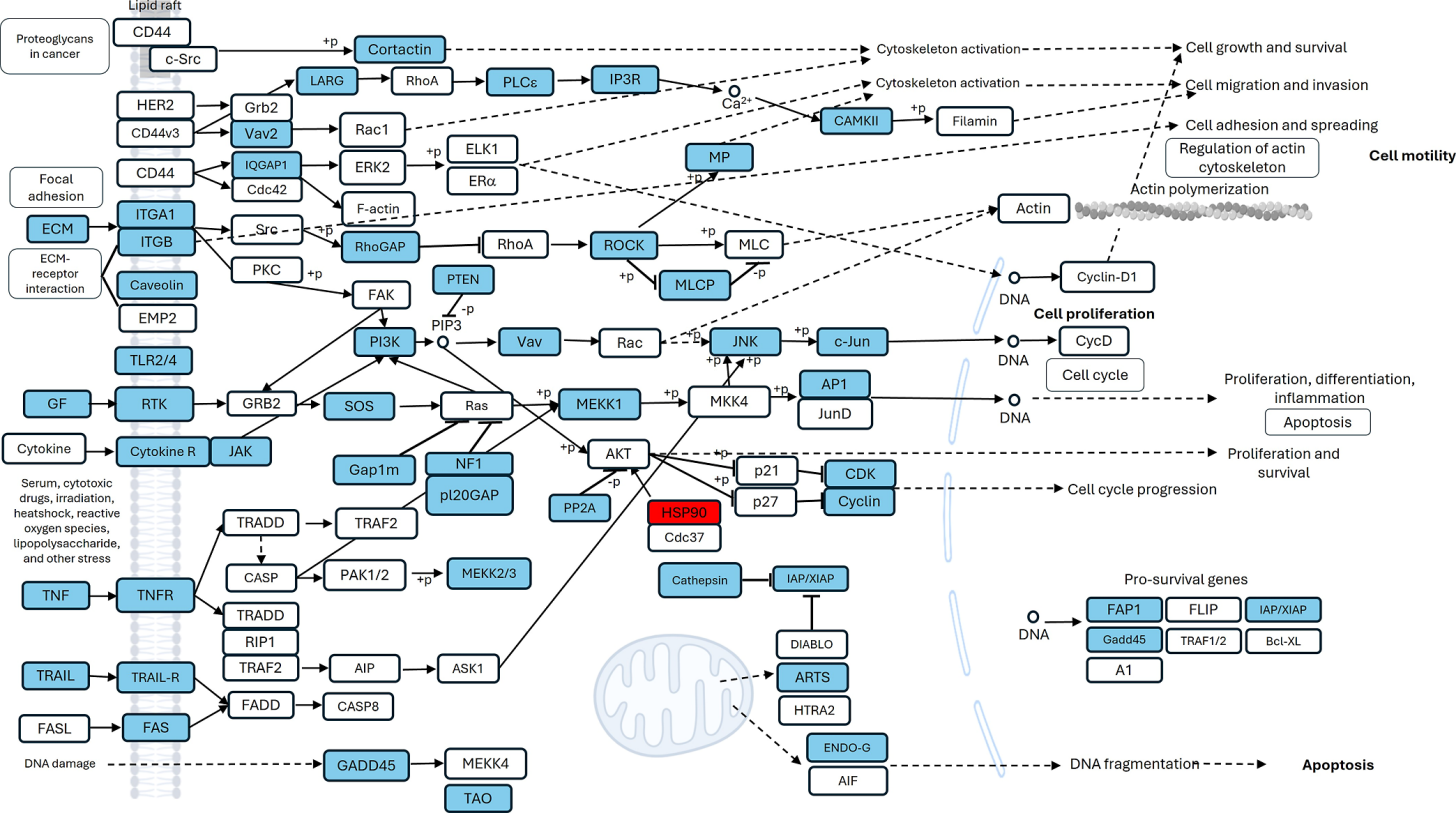
Relevant gene–gene interactions in the HCC1937 cell line were constructed on the basis of KEGG enrichment analysis by KOBAS (hsa05205, hsa04014, hsa04010, hsa04151, and hsa04510). Genes that were underexpressed (blue) or overexpressed (red) were involved in processes related to cell motility, cell proliferation and apoptosis.

**Figure 11:**
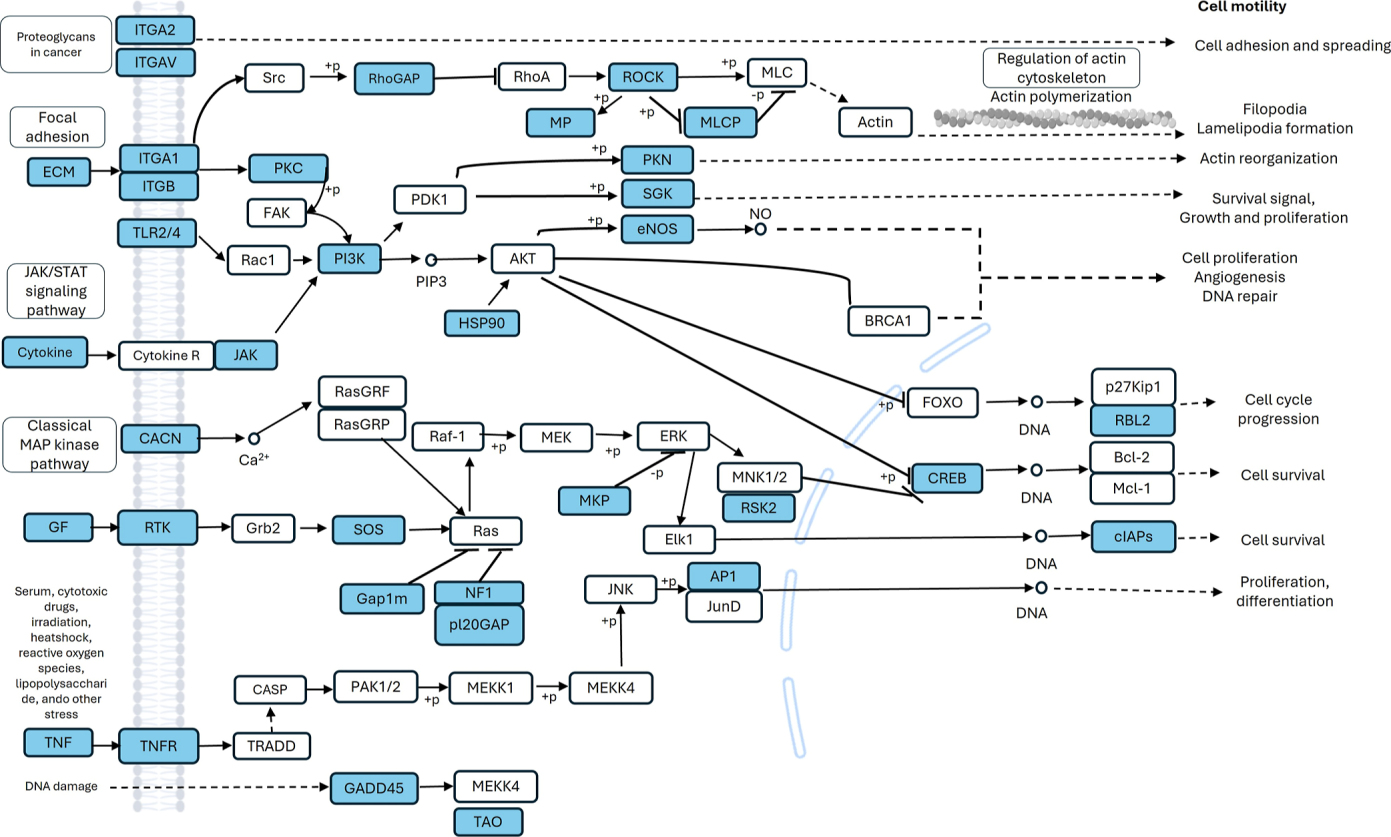
Related gene–gene interactions in the MDA-MB-231 cell line were constructed on the basis of KEGG enrichment analysis by KOBAS (hsa04510, hsa04512, hsa05205, hsa04010, and hsa04151). Genes underexpressed (blue) were involved in processes related to cell motility and cell proliferation.

## Conclusion

We provide valuable information on the molecular landscape of *K. pinnata* phytochemicals. Currently, inhibitors exist to suppress TNBC cell growth, but the use of compensatory bypass pathways makes the treatment successful, so the combined approaches have become the better option. As we showed numerous molecules in several pathways are targeted by *K. pinnata*, which could provide a great opportunity to improve the treatment strategies for its effect on tumoral cell migration and proliferation. Phosphorylation, an essential process to activate or deactivate diverse molecules, is deregulated in cancer. The present study shows that protein kinases JAK2, ROCK1, ROCK2, and PIK3CA are critical targets that can be regulated with *K. pinnata*.

## Methods

### Cell Culture

The human breast cancer line HCC1937, a breast cancer cell line representing invasive ductal carcinoma without ER or PR with a BRCA1 mutation,^32^ was cultured in RPMI 1640 medium (DMEM, Gibco, Thermo Fisher, USA). The MDA-MB-231 cell line with normal BRCA1 expression was cultured in Dulbecco’s modified Eagle’s medium (DMEM, Gibco, Thermo Fisher, USA). The media was supplemented with 100 U/mL penicillin, 100 mg/mL streptomycin, 0.25 μg/mL (Gibco, Thermo Fisher, USA) and 10% (v/v) fetal bovine serum (FBS, Biowest, Fr). The cells were incubated at 37 °C and 5% CO_2_.

### MTT Assay

To estimate the cytotoxic effects of the aqueous and ethanolic extract fractions, we performed an MTT (3-[4,5-dimethylthiazol-2-yl]-2,5-diphenyltetrazolium bromide; Sigma-Aldrich, USA) test on the cancer cell lines MDA-MB-231 and HCC1937, as we previously reported.^10^

### Wound Healing Assay

The breast cancer cells were seeded in 6-well plates until they reached 80% confluence, and we included a treated group with an aqueous extract of *K. pinnata* and a nontreated group. After confluence, the cells were scratched in a straight line via a 200 μL sterile pipet tip with a design grid to obtain the same field for each image acquisition, followed by washing with 1X PBS to remove cell debris. The scratch wound was photographed every 24 h (0–72 h) to assess cell migration toward the denuded region. Images of the cells were obtained via an inverted microscope with a 4X objective (Motic AE2000, Ca). The scratch area was determined via ImageJ software^33^ as the percentage of area reduction (wound closure). All the experiments were performed in triplicate, and the results are expressed as the means ± SEMs.

### Transwell Assay

After treatment with aqueous extract for 48 h, HCC1937 and MDA-MB-231 cells were suspended in serum-free medium and plated in the upper chambers of a 12-well companion tissue culture plate system with an 8 μm pore size polyethylene membrane (Corning, USA). HCC1937 cells were seeded at a concentration of 4 × 10^4^/well in 150 μL of RPMI, and MDA-MB-231 cells were seeded at a concentration of 9 × 10^4^/well in 150 μL of DMEM in the inset chamber. The lower chamber was filled with 800 μL of RPMI 1640 + +or DMEM containing 10% FBS. After 20 h of incubation, the cells on the upper surface of the inset chamber were removed with cotton swabs. The bottom surface of the membrane was fixed with 4% paraformaldehyde and 145 mM NaCl for 30 min and stained with crystal violet (Sigma-Aldrich). The membrane was air-dried, and the cells that traversed the membrane pores were captured via an inverted microscope with a 10× objective (Motic AE2000, Ca) and counted via ImageJ software. All the experiments were performed in triplicate, the fields were selected randomly for counting, and the results are expressed as the means ± SEMs.

### RNA-Seq Library Preparation and Data Analysis

The cells were seeded in 6-well plates and incubated until they reached 80% confluence. We included a nontreated group as a control (NT), a wound healing group (W), an aqueous extract group (AE) and a wound healing and aqueous extract group (WAE). The wound healing assay was performed as described above. At the end of treatment, total RNA was isolated from cells by using TRIzol reagent (Invitrogen, USA) according to the manufactureŕs protocol. The RNA quality was determined via an Agilent 2100 Bioanalyzer (Agilent Technologies, Santa Clara, CA). Approximately 30 ng of RNA was utilized for transcriptome library preparation per sample. Messenger RNA was purified from total RNA via poly-T oligo-attached magnetic beads. After fragmentation, first-strand cDNA was synthesized via random hexamer primers, followed by second-strand cDNA synthesis. The library was ready after end repair, A-tailing, adapter ligation, size selection, amplification, and purification. The library was checked with a Qubit and real-time PCR for quantification and a bioanalyzer for size distribution detection. The quantified libraries were pooled and sequenced on the NovaSeq (PE150) Illumina platform. The raw data were quality controlled via FastQC v0.12.1 software. The reads were evaluated for average read length, adapter content, per-sequence GC content, and sequence quality scores. The adapters and low-quality reads near the 3′ and 5′ ends were removed via fastp v0.23.4. The paired-end clean reads were aligned to the reference genome Homo_sapiens. GRCh38 using Salmon v1.10.3. Prior to differential gene expression analysis, the read counts were filtered out for quality control on the basis of the following criteria: (i) transcripts with zero counts in all samples and (ii) transcripts that were not present in at least 50% of the samples. DESeq2 v_1.44.0, which is based on a negative binomial distribution, was used to identify differentially expressed genes (DEGs). The p values were adjusted (padj) via Benjamini and Hochberg correction, which controls the false discovery rate (FDR). The principal component analysis plots and heatmaps were created via DESeq2 v1.44. Data visualization and manipulation tools were computed via R version 4.4.1.

### Analysis of Enrichment Pathways

The DEGs were identified by comparing gene expression levels between control cells and cells treated with aqueous extract on the basis of the cutoff criteria of |log2-fold-change (FC)| ≥ 2.0 and *p* value < 0.05. A Venn diagram to determine DEGs among the cell lines was drawn online with E Veen.^34^ We conducted overrepresentation analysis (ORA) and gene set enrichment analysis (GSEA) via the WebGestalt online Web site tool ( http://www.webgestalt.org/) to obtain the Gene Ontology (GO) processes. KEGG pathway analysis was conducted via KOBAS-I, an online tool that integrates the results from seven functional class scoring methods and two pathway topology tools into a single ensemble score and prioritizes the relevant biological pathways to obtain the 20 pathways that are most significant (*P* value < 0.001),^35^ and Ingenuity Pathway Analysis (IPA) software was used by Qiagen (www.ingenuity.com).

### Molecular Docking

#### Protein Preparation and Validation

Crystals for the JAK1 (4EHZ),^36^ JAK2 (3KRR),^37^ JAK3 (1YVJ),^38^ ROCK1 (6E9W),^36^ ROCK2 (6ED6),^36^ and PIK3CA (8EXU9),^36^ obtained from the Protein Data Bank were prepared in the Protein Preparation Wizard module^39^ at physiological conditions of pH 7.4 and minimized with a cutoff of a Root Mean Square Deviation (RMSD) ≤ 0.3 Å with an OPLS4 force field according to the previously reported protocol^40^ for the validation of each protein.

#### Metabolites Preparation

The molecules found in the literature were obtained from the PUBCHEM database (https://pubchem.ncbi.nlm.nih.gov/) was minimized in structure using Macromodel^39^ and brought to physiological conditions of pH 7.4 in LigPrep^39^ according to the previously reported protocol^40^ with an OPLS4 force field.

#### Molecular Docking

Molecular docking studies were performed in the Glide module^39^ with the flexible ligand technique and flexible Ser, Try, Cys, and Met residues, as well as residues at the interaction site according to the previously reported protocol^40^ with an OPLS4 force field.

### Statistical Analysis

Data are presented as mean ± SEM. All statistical analyses were performed using GraphPad Prism 10.2.2 (GraphPad Software, Inc., San Diego, CA, USA). The results were considered significant for *p*-value <0.05.

## Supplementary Material



## Data Availability

The original contributions presented in the study are publicly available. This data can be found in the National Center for Biotechnology Information in the Gene Expression Omnibus (GEO) repository, the project number is GSE287572.

## Notes

Figure S1 Table 1S Table 2S Table 3S Table 4S (PDF)
