# Supplementary material for: Transcriptome Analysis of Triple-Negative HCC1937 and MDA-MB-231 Breast Cancer Cells Treated with Kalanchoe pinnata Revealed the Regulation of Migration and Invasion via the Downregulation of the Genes JAK2, ROCK1 and ROCK2
Source: ACS Omega. 2025 Jul 9;10(28):31187–200. doi: 10.1021/acsomega.5c05895 (PMC12290928; doi:10.1021/acsomega.5c05895)
Supplement: Supplementary file 1 [file ao5c05895_si_001.pdf]

Transcriptome analysis of triple-negative HCC1937 and MDA-MB-231 breast cancer cells treated with *Kalanchoe pinnata* revealed the regulation of migration and invasion via the downregulation of the genes *JAK2*, *ROCK1* and *ROCK2*.

Carlos Alvizo-Rodríguez [1], Alan Carrasco-Carballo[2,3], Uriel López-Vázquez [1], Georgina Hernández-Montes[4], Marta E. Hernández-Caballero [1*]

1 Facultad de Medicina, Biomedicina, Benemérita Universidad Autónoma de Puebla. 13 sur 2702 Col. Volcanes, C.P. 72410. Puebla, México.

2 Laboratorio de Elucidación y Síntesis en Química Orgánica, Instituto de Ciencias, Benemérita Universidad Autónoma de Puebla, Puebla, México

3 Secretaría de Ciencia, Humanidades, Tecnología e Innovación (SECIHTI) Av. Insurgentes Sur 1582, Col. Crédito Constructor, Demarcación Territorial Benito Juárez. CP 03940, Ciudad de México.

4 Coordinación de la Investigación Científica, UNAM - Red de Apoyo a la Investigación. Vasco de Quiroga 15, Sección XVI, Tlalpan C.P. 14000 Ciudad de México, México.

* Correspondence: elena.hernandezcab@ correo.buap.mx

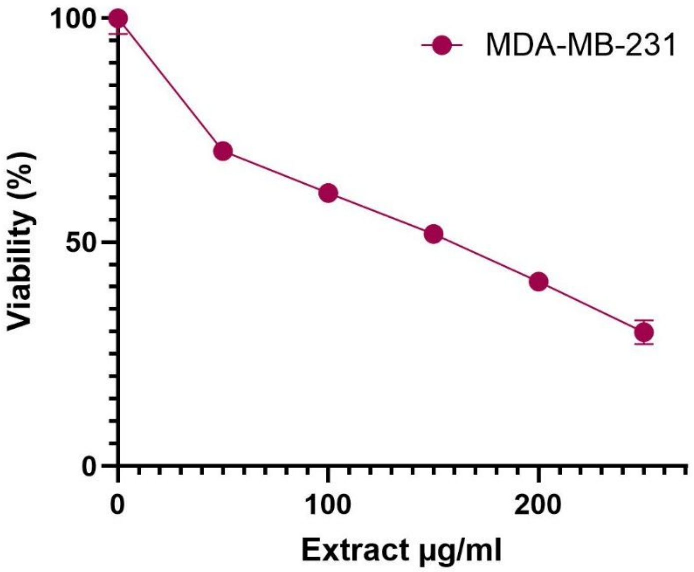

Figure 1S. Cytotoxic effects of aqueous extract of *K. pinnata* cells after 72 h of treatment in MDA-MB-231 cells.

MTT assay

MDA-MB-231 cells		
Extract μg/mL	Viability (%)	SE
0	100	3.516807
50	70.316153	1.032757
100	60.963708	1.146491
150	51.814578	1.896109
200	41.201586	0.941599
250	29.927824	2.650064

Table 1S. ORA analysis of HCC1937 and MDA-MB-231 breast cancer cells treated with *K. pinnata* aqueous extract.

HCC1937						
**Biological process**						
**Gene Set**	**Description**	**Size**	**ES**	**NES**	**P Value**	**FDR**
GO:0007059	Chromosome segregation	433	42.165	1.8973	1.1932e-8	0.000010011
GO:0044782	Cilium organization	435	42.359	1.8178	1.4444e-7	0.000060593
GO:0051383	Kinetochore organization	21	2.0449	5.3791	0.0000010085	0.00028203
GO:0048285	Organelle fission	490	47.715	1.6347	0.0000084276	0.0014979
GO:0140694	Non-membrane-bounded organelle assembly	404	39.341	1.7031	0.0000093446	0.0014979
GO:0031023	Microtubule organizing center organization	154	14.996	2.2006	0.000010712	0.0014979
GO:1901987	Regulation of cell cycle phase transition	420	40.899	1.6382	0.000033642	0.0040323
GO:0045787	Positive regulation of cell cycle	313	30.479	1.7389	0.000044263	0.0046420
GO:0051321	Meiotic cell cycle	287	27.947	1.7533	0.000068467	0.0063827
GO:0033044	Regulation of chromosome organization	245	23.858	1.8024	0.000098185	0.0082377
**Cellular Component**						
**Gene Set**	**Description**	**Size**	**ES**	**NES**	**P Value**	**FDR**
GO:0005814	Centriole	157	15.707	2.4192	1.6885e-7	0.000031913
GO:0098687	Chromosomal region	385	38.518	1.7654	0.0000019031	0.00017984
GO:0000793	Condensed chromosome	280	28.013	1.8206	0.000015572	0.00095532
GO:0000242	Pericentriolar material	22	2.2010	4.5433	0.000020218	0.00095532
GO:0034451	Centriolar satellite	125	12.506	2.2389	0.000032687	0.0012356
GO:0036064	Ciliary basal body	172	17.208	1.9758	0.000076939	0.0024236
GO:0098858	Actin-based cell projection	225	22.511	1.7325	0.00043515	0.011749
GO:0005819	Spindle	425	42.520	1.5052	0.00052082	0.012304
GO:0016605	PML body	107	10.705	2.0551	0.00078145	0.016410
GO:0030990	Intraciliary transport particle	26	2.6012	3.0755	0.0029518	0.051271
**Molecular Function**						
**Gene Set**	**Description**	**Size**	**ES**	**NES**	**P Value**	**FDR**

GO:0140097	Catalytic activity, acting on DNA	241	22.853	2.2754	1.0777e-8	0.0000029960
GO:0015631	Tubulin binding	381	36.128	1.9375	4.0605e-8	0.0000056441
GO:0060589	Nucleoside-triphosphatase regulator activity	483	45.800	1.7249	8.6678e-7	0.000080322
GO:0004386	Helicase activity	154	14.603	2.2598	0.0000060007	0.00041705
GO:0016887	ATP hydrolysis activity	404	38.309	1.6706	0.000026747	0.0014871
GO:0003774	Cytoskeletal motor activity	112	10.620	2.0715	0.00073450	0.034032
GO:0106310	Protein serine kinase activity	356	33.758	1.5404	0.0010638	0.042247
GO:0019783	Ubiquitin-like protein peptidase activity	128	12.138	1.8949	0.0019799	0.068803
GO:0051020	Gtpase binding	315	29.870	1.5065	0.0034430	0.10635
GO:0008234	Cysteine-type peptidase activity	183	17.353	1.6136	0.0075580	0.20072
			**MDA-MB-231**			
**Biological process**						
**Gene Set**	**Description**	**Size**	**ES**	**NES**	**P Value**	**FDR**
GO:0044782	Cilium organization	435	34.424	2.1496	2.1145e-10	1.7740e-7
GO:0007018	Microtubule-based movement	425	33.633	1.9921	3.5709e-8	0.000014980
GO:0099111	Microtubule-based transport	214	16.935	2.3029	7.0337e-7	0.00019671
GO:0140694	Non-membrane-bounded organelle assembly	404	31.971	1.8767	0.0000014585	0.00030593
GO:0022613	Ribonucleoprotein complex biogenesis	450	35.611	1.7410	0.000011682	0.0017354
GO:0007059	Chromosome segregation	433	34.266	1.7510	0.000013525	0.0017354
GO:0051383	Kinetochore organization	21	1.6619	5.4156	0.000014479	0.0017354
GO:0008380	RNA splicing	446	35.295	1.7283	0.000017221	0.0018060
GO:0030705	Cytoskeleton-dependent intracellular transport	210	16.619	2.1061	0.000019833	0.0018489
GO:0007051	Spindle organization	209	16.539	1.9952	0.00010113	0.0084845
**Cellular Component**						
**Gene Set**	**Description**	**Size**	**ES**	**NES**	**P Value**	**FDR**
GO:0005814	Centriole	157	13.527	2.8831	8.2961e-10	1.5680e-7
GO:0034451	Centriolar satellite	125	10.770	2.6927	5.8400e-7	0.000055188

GO:0000793	Condensed chromosome	280	24.125	1.8239	0.000063110	0.0039759
GO:0098687	Chromosomal region	385	33.171	1.6279	0.00022018	0.010404
GO:0036064	Ciliary basal body	172	14.819	1.8894	0.00074461	0.028146
GO:0090734	Site of DNA damage	121	10.425	2.0143	0.0014510	0.043754
GO:0016605	PML body	107	9.2191	2.0609	0.0018209	0.043754
GO:0000242	Pericentriolar material	22	1.8955	3.6929	0.0018520	0.043754
GO:0005819	Spindle	425	36.618	1.4747	0.0023089	0.048488
GO:0005801	Cis-Golgi network	66	5.6865	2.2861	0.0036722	0.067296
**Molecular Function**						
**Gene Set**	**Description**	**Size**	**ES**	**NES**	**P Value**	**FDR**
GO:0016887	ATP hydrolysis activity	404	30.803	2.1751	8.9303e-10	1.7613e-7
GO:0015631	Tubulin binding	381	29.049	2.2032	1.2671e-9	1.7613e-7
GO:0004386	Helicase activity	154	11.742	2.8957	1.2076e-8	0.0000011190
GO:0140097	Catalytic activity, acting on DNA	241	18.375	2.2313	8.7423e-7	0.000060759
GO:0003774	Cytoskeletal motor activity	112	8.5393	2.6934	0.0000099327	0.00055226
GO:0042393	Histone binding	244	18.604	1.8276	0.00044027	0.020399
GO:0060589	Nucleoside-triphosphatase regulator activity	483	36.826	1.5478	0.00060468	0.024015
GO:0019783	Ubiquitin-like protein peptidase activity	128	9.7592	2.0493	0.0016108	0.055974
GO:0003779	Actin binding	431	32.861	1.4911	0.0030790	0.095108
GO:0098631	Cell adhesion mediator activity	66	5.0321	2.3847	0.0038159	0.10608

ES: Enrichment Score; NES: Normalized Enrichment Score; FDR: Significance level

Table 2S. GSEA analysis of HCC1937 breast cancer cells treated with *K. pinnata* aqueous extract.

**Down-regulated genes**						
**Biological process**						
Gene Set	Description	Size	ES	NES	P Value	FDR
GO:0098742	Cell-cell adhesion via plasma-membrane adhesion molecules	25	-0.57634	-2.6921	<2.2e-16	<2.2e-16

GO:0007586	Digestion	9	-0.69018	-2.2064	<2.2e-16	0.069579
GO:0009593	Detection of chemical stimulus	10	-0.64357	-2.13	0.002642	0.094908
GO:0008037	Cell recognition	12	-0.57747	-2.0446	0.001295	0.16525
GO:0099177	Regulation of trans-synaptic signaling	39	-0.37719	-1.9997	0.002174	0.19684
GO:0007051	Spindle organization	36	-0.38426	-1.9677	0.001092	0.21698
GO:0099565	Chemical synaptic transmission, postsynaptic	6	-0.70908	-1.9148	0.001468	0.28904
GO:0051383	Kinetochore organization	11	-0.54001	-1.83	0.01087	0.48797
GO:0008380	RNA splicing	54	0.17158	1.6109	0.021739	0.50542
GO:0016042	Lipid catabolic process	28	0.2311	1.5513	0.065421	0.50614
GO:0007159	Leukocyte cell-cell adhesion	28	0.23273	1.5908	0.034483	0.51286
GO:0006260	DNA replication	44	0.17775	1.5624	0.041096	0.51307
GO:0007606	Sensory perception of chemical stimulus	10	-0.53512	-1.7624	0.010554	0.52947
GO:0003012	Muscle system process	39	-0.34357	-1.8018	0.012022	0.53934
GO:0031529	Ruffle organization	5	0.55648	1.5629	0.047619	0.54566
GO:0007264	Small gtpase mediated signal transduction	61	0.15918	1.6301	0.029412	0.54701
GO:0035264	Multicellular organism growth	16	0.31634	1.6109	0.040404	0.54754
GO:0050727	Regulation of inflammatory response	26	0.22787	1.5101	0.058065	0.5686
GO:0042176	Regulation of protein catabolic process	32	0.2005	1.4721	0.077778	0.57484
GO:0002244	Hematopoietic progenitor cell differentiation	19	0.25367	1.4754	0.080925	0.59468
**Cellular Component**						
Gene Set	Description	Size	ES	NES	P Value	FDR
GO:0000151	Ubiquitin ligase complex	22	0.30194	1.777	0.006494	0.23901
GO:0015030	Cajal body	7	0.54884	1.8442	0.010101	0.26087
GO:0005667	Transcription regulator complex	40	0.19326	1.6014	0.041096	0.27037
GO:0099023	Vesicle tethering complex	8	0.48553	1.6822	0.02509	0.28011
GO:0030055	Cell-substrate junction	28	0.23127	1.6073	0.027027	0.31732
GO:1902493	Acetyltransferase complex	9	0.52047	1.9043	0.00365	0.3521
GO:0005912	Adherens junction	14	0.31875	1.4925	0.066351	0.3855
GO:0098982	GABA-ergic synapse	5	0.49872	1.3917	0.11144	0.45743
GO:0042579	Microbody	14	0.29337	1.4077	0.098592	0.48361
GO:0019898	Extrinsic component of membrane	25	0.1762	1.1889	0.1938	0.52862
GO:0031045	Dense core granule	6	-0.63841	-1.7116	0.018895	0.60078
GO:0005814	Centriole	37	-0.29806	-1.5598	0.053552	0.75876
GO:0005775	Vacuolar lumen	8	-0.57256	-1.7307	0.021368	0.79269
GO:0031514	Motile cilium	23	-0.24102	-1.0665	0.37123	0.85655
GO:0045111	Intermediate filament cytoskeleton	13	-0.31891	-1.1664	0.2834	0.85923
GO:0098687	Chromosomal region	66	-0.18122	-1.082	0.38413	0.86103
GO:0098862	Cluster of actin-based cell projections	23	-0.24549	-1.0858	0.37529	0.87426

GO:0032432	Actin filament bundle	11	-0.31637	-1.067	0.38608	0.87679
GO:0099572	Postsynaptic specialization	32	-0.22303	-1.1028	0.35327	0.87716
GO:0097730	Non-motile cilium	24	-0.24465	-1.0934	0.36396	0.8772
**Molecular Function**						
Gene Set	Description	Size	ES	NES	P Value	FDR
GO:0001664	G protein-coupled receptor binding	11	0.49126	2.0543	0.004032	0.16907
GO:0016298	Lipase activity	10	0.47843	1.8571	0.022059	0.19264
GO:0003712	Transcription coregulator activity	53	0.18716	1.7419	0.037037	0.21457
GO:0140678	Molecular function inhibitor activity	41	0.22501	1.8956	0.011765	0.21826
GO:0051219	Phosphoprotein binding	7	0.52176	1.7007	0.04698	0.22005
GO:0001216	DNA-binding transcription activator activity	24	0.25601	1.6014	0.055944	0.23568
GO:0140272	Exogenous protein binding	5	0.56675	1.5752	0.050898	0.23977
GO:0001221	Transcription coregulator binding	8	0.43151	1.5494	0.060284	0.24271
GO:0019208	Phosphatase regulator activity	12	0.37137	1.6161	0.031674	0.24496
GO:0001217	DNA-binding transcription repressor activity	32	0.24696	1.7494	0.018349	0.25976
GO:0043178	Alcohol binding	10	-0.53115	-1.7376	0.018792	0.37599
GO:0005518	Collagen binding	7	-0.57671	-1.6334	0.029455	0.38182
GO:0016705	Oxidoreductase activity, acting on paired donors, with incorporation or reduction of molecular oxygen	11	-0.50003	-1.6981	0.02046	0.39417
GO:0008237	Metallopeptidase activity	18	-0.3843	-1.5725	0.049818	0.40139
GO:0003697	Single-stranded DNA binding	15	-0.4117	-1.5298	0.061069	0.40168
GO:0070840	Dynein complex binding	5	-0.61387	-1.5428	0.045942	0.4026
GO:0016887	ATP hydrolysis activity	60	-0.26753	-1.5543	0.03277	0.40918
GO:0005216	Monoatomic ion channel activity	30	-0.34375	-1.6622	0.033372	0.41702
GO:0019840	Isoprenoid binding	6	-0.58015	-1.581	0.043541	0.41959
GO:0030276	Clathrin binding	5	-0.64717	-1.6345	0.02099	0.43322
**Up-regulated genes**						
**Biological process**						
Gene Set	Description	Size	ES	NES	P Value	FDR
GO:0072522	Purine-containing compound biosynthetic process	6	-0.72043	-2.1705	<2.2e-16	0.11291
GO:0034470	Ncrna processing	10	-0.50909	-2.0532	0.008889	0.11291
GO:0022613	Ribonucleoprotein complex biogenesis	9	-0.50725	-1.8979	0.012346	0.2027
GO:0072523	Purine-containing compound catabolic process	6	-0.58781	-1.7794	0.027682	0.242
GO:1901293	Nucleoside phosphate biosynthetic process	7	-0.55659	-1.7949	0.011719	0.2836
GO:0016072	Rrna metabolic process	6	-0.50179	-1.5513	0.067114	0.34832

GO:0046434	Organophosphate catabolic process	9	-0.43116	-1.6619	0.039474	0.35724
GO:0019693	Ribose phosphate metabolic process	10	-0.40364	-1.5627	0.03139	0.37014
GO:1901136	Carbohydrate derivative catabolic process	7	-0.4964	-1.6141	0.050193	0.37987
GO:0072524	Pyridine-containing compound metabolic process	6	-0.51254	-1.5817	0.064626	0.38506
GO:0002237	Response to molecule of bacterial origin	13	0.55171	1.8747	0.003708	0.39757
GO:0042742	Defense response to bacterium	10	0.57963	1.7692	0.010363	0.40194
GO:1901617	Organic hydroxy compound biosynthetic process	8	0.61166	1.7825	0.005384	0.45091
GO:0018958	Phenol-containing compound metabolic process	6	0.69933	1.8155	0.006964	0.4569
GO:0042330	Taxis	9	0.57557	1.7197	0.018519	0.48188
GO:0042445	Hormone metabolic process	8	0.6641	1.8925	0.001351	0.66817
GO:0008202	Steroid metabolic process	10	0.53014	1.6406	0.022222	0.72199
GO:0010506	Regulation of autophagy	5	0.59358	1.4639	0.069264	0.74509
GO:0098742	Cell-cell adhesion via plasma-membrane adhesion molecules	6	0.58275	1.4645	0.08227	0.78123
GO:0007249	Canonical NF-kappab signal transduction	5	0.5869	1.4307	0.071535	0.80366

Cellular Component

Gene Set	Description	Size	ES	NES	P Value	FDR
GO:1990351	Transporter complex	7	0.68564	1.8879	0.006944	0.030271
GO:0043235	Receptor complex	6	0.7276	1.8934	<2.2e-16	0.057033
GO:0005759	Mitochondrial matrix	8	-0.48375	-1.7096	0.030303	0.13636
GO:0097060	Synaptic membrane	9	0.53173	1.6027	0.030872	0.18338
GO:0031253	Cell projection membrane	11	-0.37591	-1.5121	0.069307	0.20976
GO:0098984	Neuron to neuron synapse	5	0.61561	1.5131	0.08	0.24305
GO:0098798	Mitochondrial protein-containing complex	5	-0.63929	-1.7152	0.040881	0.26741
GO:0043025	Neuronal cell body	10	0.45397	1.4134	0.11041	0.27288
GO:0005769	Early endosome	12	0.44465	1.4483	0.095178	0.27551
GO:0005743	Mitochondrial inner membrane	13	-0.29127	-1.2873	0.16176	0.38348
GO:0005635	Nuclear envelope	5	-0.40714	-1.1271	0.31461	0.53401
GO:0001533	Cornified envelope	5	0.41786	1.0285	0.41691	0.61652
GO:0031983	Vesicle lumen	5	0.48859	1.1672	0.29438	0.65231
GO:0030139	Endocytic vesicle	12	0.31177	1.0321	0.42892	0.67264
GO:0031252	Cell leading edge	7	-0.30935	-0.9857	0.44275	0.6745
GO:0045111	Intermediate filament cytoskeleton	6	0.40599	1.0464	0.39451	0.72145
GO:0062023	Collagen-containing extracellular matrix	9	-0.17029	-0.6253	0.95279	0.92328
GO:0005874	Microtubule	5	-0.23929	-0.6558	0.88963	1

GO:0019867	Outer membrane	6	-0.21147	-0.6389	0.91166	1
**Molecular Function**						
Gene Set	Description	Size	ES	NES	P Value	FDR
**GO:0005216**	monoatomic ion channel activity	8	0.71932	2.0576	0.001357	0.011483
**GO:0022836**	gated channel activity	7	0.69798	1.9336	0.001351	0.024402
**GO:0060589**	nucleoside-triphosphatase regulator activity	10	-0.35207	-1.3586	0.11521	0.28647
**GO:0045296**	cadherin binding	6	-0.46321	-1.4064	0.09894	0.29402
**GO:0042578**	phosphoric ester hydrolase activity	11	-0.40733	-1.6827	0.020202	0.31668
**GO:0016741**	transferase activity, transferring one-carbon groups	5	-0.52143	-1.4511	0.10423	0.32675
**GO:0140098**	catalytic activity, acting on RNA	7	-0.46403	-1.5149	0.059259	0.37208
**GO:0004518**	nuclease activity	5	-0.40714	-1.112	0.32509	0.42033
**GO:0140097**	catalytic activity, acting on DNA	5	-0.40714	-1.1196	0.3	0.47095
**GO:0005319**	lipid transporter activity	7	-0.34881	-1.1604	0.25362	0.4864
**GO:0051020**	GTPase binding	6	-0.34711	-1.0271	0.40678	0.49374
**GO:0016755**	aminoacyltransferase activity	7	0.47285	1.2946	0.17892	0.50611
**GO:0031072**	heat shock protein binding	5	0.5733	1.3736	0.12408	0.53477
**GO:0005506**	iron ion binding	6	0.5059	1.3022	0.17373	0.55323
**GO:0044389**	ubiquitin-like protein ligase binding	5	0.53774	1.322	0.14559	0.58209
**GO:0001217**	DNA-binding transcription repressor activity	8	0.40428	1.1729	0.28743	0.59872
**GO:0030546**	signaling receptor activator activity	23	0.33611	1.3826	0.13348	0.61569
**GO:0016209**	antioxidant activity	5	0.4224	1.0486	0.41104	0.61683
**GO:0033218**	amide binding	10	0.32602	1.0248	0.44258	0.62121
**GO:0022804**	active transmembrane transporter activity	7	-0.2554	-0.82933	0.66797	0.66857

ES: Enrichment Score; NES: Normalized Enrichment Score; FDR: Significance level

Table 3S. GSEA analysis of MDA-MB-231 breast cancer cells treated with *K. pinnata* aqueous extract

**Down-regulated genes**						
**Biological process**						
**Gene Set**	**Description**	**Size**	**ES**	**NES**	**P Value**	**FDR**
GO:0098742	Cell-cell adhesion via plasma-membrane adhesion molecules	34	-0.44063	-2.193	0.001103	0.11503
GO:0051383	Kinetochore organization	9	-0.65254	-2.0569	0.001383	0.22876
GO:0051321	Meiotic cell cycle	32	-0.39397	-1.8857	0.004381	0.28368
GO:0033044	Regulation of chromosome organization	35	-0.38776	-1.9426	0.002181	0.30617
GO:0065004	Protein-DNA complex assembly	21	-0.44271	-1.8929	0.007169	0.30978
GO:0007059	Chromosome segregation	58	-0.33182	-1.8939	0.002073	0.36914
GO:0098727	Maintenance of cell number	19	-0.4728	-1.945	0.003663	0.40044

GO:0031503	Protein-containing complex localization	22	-0.42298	-1.8222	0.006889	0.40974
GO:0097722	Sperm motility	19	-0.43878	-1.7818	0.009524	0.49549
GO:0008277	Regulation of G protein-coupled receptor signaling pathway	6	-0.63604	-1.7363	0.008511	0.55565
GO:0070269	Pyroptosis	6	0.49183	1.5303	0.057239	0.79858
GO:0051051	Negative regulation of transport	27	0.17449	1.1899	0.22727	0.8033
GO:0007159	Leukocyte cell-cell adhesion	23	0.19056	1.1956	0.18939	0.8053
GO:0034341	Response to type II interferon	9	0.32135	1.1988	0.23045	0.81507
GO:0015980	Energy derivation by oxidation of organic compounds	10	0.3123	1.2034	0.21311	0.82019
GO:0006520	Amino acid metabolic process	9	0.32784	1.2402	0.1769	0.83055
GO:0097193	Intrinsic apoptotic signaling pathway	23	0.19589	1.2049	0.21805	0.83378
GO:0044282	Small molecule catabolic process	9	0.40158	1.5394	0.05948	0.83663
GO:0006354	DNA-templated transcription elongation	6	0.57271	1.714	0.023891	0.8396
GO:0042180	Cellular ketone metabolic process	10	0.30984	1.2078	0.22458	0.84542
**Cellular component**						
**Gene Set**	**Description**	**Size**	**ES**	**NES**	**P Value**	**FDR**
GO:0098687	Chromosomal region	49	-0.40963	-2.2255	0.001059	0.020653
GO:0000793	Condensed chromosome	38	-0.42227	-2.1266	0.001091	0.029012
GO:0005874	Microtubule	54	-0.333	-1.8487	0.004215	0.2062
GO:0005635	Nuclear envelope	35	-0.3416	-1.6935	0.01634	0.37155
GO:0005814	Centriole	38	-0.33817	-1.7264	0.017149	0.37248
GO:0000792	Heterochromatin	11	-0.46533	-1.617	0.04106	0.37347
GO:0005819	Spindle	51	-0.30139	-1.6498	0.027112	0.40649
GO:0000228	Nuclear chromosome	24	-0.36522	-1.6207	0.033487	0.41839
GO:0005875	Microtubule associated complex	23	-0.34491	-1.532	0.068287	0.54024
GO:0099568	Cytoplasmic region	31	-0.30635	-1.498	0.091422	0.56661
GO:0000151	Ubiquitin ligase complex	16	0.28575	1.462	0.067485	0.90547
GO:0016459	Myosin complex	6	0.36275	1.0879	0.35762	0.94828
GO:0016234	Inclusion body	8	0.13632	0.47782	0.98966	0.99624
GO:0101002	Ficolin-1-rich granule	11	0.17309	0.72568	0.82627	0.99839
GO:0001650	Fibrillar center	15	0.30782	1.526	0.044944	1
GO:0005667	Transcription regulator complex	29	0.18984	1.3044	0.18269	1
GO:0005788	Endoplasmic reticulum lumen	17	0.22747	1.2037	0.19444	1
GO:0005759	Mitochondrial matrix	11	0.27183	1.1379	0.24424	1
GO:0072562	Blood microparticle	6	0.35131	1.1132	0.31475	1
GO:0005912	Adherens junction	10	0.27917	1.0893	0.34025	1
**Molecular Function**						
**Gene Set**	**Description**	**Size**	**ES**	**NES**	**P Value**	**FDR**
GO:0003697	Single-stranded DNA binding	11	-0.61663	-2.0984	<2.2e-16	0.073801
GO:0070840	Dynein complex binding	5	-0.7672	-1.9445	0.002924	0.13925

GO:0015631	Tubulin binding	62	-0.2772	-1.6448	0.023711	0.65894
GO:0140098	Catalytic activity, acting on RNA	17	0.22154	1.1651	0.27219	0.74583
GO:0016887	ATP hydrolysis activity	60	-0.28873	-1.6671	0.017544	0.76436
GO:0005216	Monoatomic ion channel activity	23	0.28915	1.7693	<2.2e-16	0.79238
GO:0043177	Organic acid binding	7	0.35492	1.1684	0.25175	0.79371
GO:0001216	DNA-binding transcription activator activity	29	0.17253	1.1904	0.22414	0.79657
GO:0008186	ATP-dependent activity, acting on RNA	8	0.30864	1.089	0.32971	0.80142
GO:0016741	Transferase activity, transferring one-carbon groups	8	0.30524	1.1039	0.2898	0.80943
GO:0003725	Double-stranded RNA binding	5	0.50995	1.4119	0.11513	0.8119
GO:0002020	Protease binding	6	0.34423	1.0613	0.37855	0.82372
GO:0008234	Cysteine-type peptidase activity	21	0.20182	1.1995	0.18543	0.84027
GO:0008509	Monoatomic anion transmembrane transporter activity	10	0.24918	1.0205	0.42259	0.84334
GO:0031072	Heat shock protein binding	11	-0.14081	-0.4815	0.98248	0.98303
GO:0042162	Telomeric DNA binding	6	-0.18722	-0.5005	0.97167	0.98777
GO:0005543	Phospholipid binding	27	-0.10853	-0.5087	0.96158	0.99574
GO:0051087	Protein-folding chaperone binding	8	-0.17332	-0.5256	0.98082	0.99948
GO:0005539	Glycosaminoglycan binding	12	-0.4303	-1.5275	0.049872	1
GO:0030546	Signaling receptor activator activity	18	-0.32672	-1.3253	0.17324	1
			**Up-regulated genes**			
**Biological process**						
**Gene Set**	**Description**	**Size**	**ES**	**NES**	**P Value**	**FDR**
GO:0016072	rRNA metabolic process	25	-0.26569	-1.6355	0.044335	0.22548
GO:0051169	Nuclear transport	5	-0.61149	-1.6494	0.037037	0.23609
GO:0072594	Establishment of protein localization to organelle	7	-0.5034	-1.658	0.035714	0.25806
GO:1903050	Regulation of proteolysis involved in protein catabolic process	6	-0.56464	-1.6859	0.042553	0.26391
GO:0022613	Ribonucleoprotein complex biogenesis	36	-0.22823	-1.7024	0.013986	0.29627
GO:0048568	Embryonic organ development	5	-0.56081	-1.5193	0.083799	0.31725
GO:0022900	Electron transport chain	6	0.70665	1.9384	0.002907	0.32891
GO:0010506	Regulation of autophagy	7	-0.46032	-1.4895	0.081356	0.33319
GO:0006605	Protein targeting	6	-0.50169	-1.5252	0.071023	0.33888
GO:0043161	Proteasome-mediated ubiquitin-dependent protein catabolic process	7	-0.53401	-1.7059	0.029674	0.3632
GO:0031331	Positive regulation of cellular catabolic process	5	-0.68243	-1.823	0.015106	0.37885
GO:0008544	Epidermis development	9	0.23288	0.74678	0.77557	0.9239
GO:0048511	Rhythmic process	6	0.27289	0.7387	0.769	0.92467

GO:0034329	Cell junction assembly	5	0.26431	0.66868	0.87348	0.92684
GO:0009314	Response to radiation	5	0.29896	0.75242	0.77429	0.92742
GO:0043269	Regulation of monoatomic ion transport	7	0.24767	0.72738	0.81654	0.9298
GO:0140694	Non-membrane-bounded organelle assembly	12	0.21009	0.75534	0.75034	0.93466
GO:0030522	Intracellular receptor signaling pathway	6	0.24407	0.67066	0.86538	0.93524
GO:0019216	Regulation of lipid metabolic process	6	0.25104	0.68871	0.83333	0.93627
GO:0007389	Pattern specification process	8	0.24915	0.76169	0.7511	0.93712
**Cellular component**						
**Gene Set**	**Description**	**Size**	**ES**	**NES**	**P Value**	**FDR**
GO:0045111	Intermediate filament cytoskeleton	7	0.54015	1.5699	0.050287	0.26323
GO:0070469	Respirasome	5	0.6802	1.7254	0.015408	0.30365
GO:1990351	Transporter complex	11	0.45702	1.5805	0.034899	0.3717
GO:0005840	Ribosome	5	-0.48311	-1.3054	0.18436	0.53529
GO:0005770	Late endosome	8	-0.39249	-1.364	0.14103	0.65069
GO:0016607	Nuclear speck	5	0.52034	1.3227	0.14241	0.65198
GO:0000228	Nuclear chromosome	6	0.3967	1.0699	0.38244	0.70246
GO:0043292	Contractile fiber	5	0.48357	1.2418	0.18361	0.71673
GO:0030684	Preribosome	14	-0.32404	-1.5007	0.082988	0.71784
GO:0098687	Chromosomal region	5	0.39769	1.0077	0.42457	0.74867
GO:0005774	Vacuolar membrane	9	-0.22425	-0.8244	0.64263	0.75155
GO:0000793	Condensed chromosome	6	0.39953	1.085	0.35426	0.75671
GO:0043025	Neuronal cell body	5	0.44439	1.1143	0.31866	0.78889
GO:0045178	Basal part of cell	5	-0.30068	-0.8263	0.66667	0.84187
GO:0005743	Mitochondrial inner membrane	18	0.27471	1.1359	0.31202	0.86004
GO:0005681	Spliceosomal complex	6	-0.28814	-0.8679	0.6457	0.87183
GO:0061695	Transferase complex, transferring phosphorus-containing groups	6	-0.19661	-0.5834	0.95238	0.95797
GO:0043235	Receptor complex	5	-0.32432	-0.8909	0.5873	0.96388
GO:0030139	Endocytic vesicle	5	-0.37394	-1.0411	0.37467	0.96851
GO:0005635	Nuclear envelope	9	-0.23978	-0.9030	0.51572	1
GO:0005743	Mitochondrial inner membrane	25	0.51894	3.238	<2.2e-16	<2.2e-16
GO:0030684	Preribosome	18	0.57205	2.999	<2.2e-16	<2.2e-16
GO:0005759	Mitochondrial matrix	26	0.43872	2.8435	<2.2e-16	<2.2e-16
GO:0005874	Microtubule	54	-0.41462	-2.4148	<2.2e-16	<2.2e-16
GO:0098687	Chromosomal region	54	-0.42009	-2.4629	<2.2e-16	<2.2e-16
GO:0005819	Spindle	54	-0.37979	-2.1981	<2.2e-16	0.006941
GO:0000793	Condensed chromosome	44	-0.39859	-2.2112	<2.2e-16	0.007831
GO:0005814	Centriole	39	-0.39556	-2.1162	<2.2e-16	0.015591
GO:0031514	Motile cilium	33	-0.39288	-1.9592	0.006113	0.04531
GO:0000792	Heterochromatin	11	-0.54672	-1.8599	0.001437	0.089971
**Molecular Function**						

Gene Set	Description	Size	ES	NES	P Value	FDR
GO:0009055	Electron transfer activity	5	0.6802	1.7418	0.012214	0.16984
GO:0030546	Signaling receptor activator activity	12	0.51086	1.816	0.013755	0.20211
GO:0005543	Phospholipid binding	5	0.62838	1.5587	0.046012	0.34591
GO:0016755	Aminoacyltransferase activity	5	-0.48311	-1.3362	0.14448	0.43393
GO:0042826	Histone deacetylase binding	5	-0.48986	-1.351	0.13115	0.51438
GO:0140993	Histone modifying activity	5	-0.51352	-1.4134	0.09116	0.55546
GO:0016705	Oxidoreductase activity, acting on paired donors, with incorporation or reduction of molecular oxygen	6	-0.48956	-1.4822	0.088235	0.63825
GO:0042393	Histone binding	5	-0.59459	-1.621	0.046322	0.68193
GO:0043021	Ribonucleoprotein complex binding	5	-0.26014	-0.7088	0.83979	0.88856
GO:0008514	Organic anion transmembrane transporter activity	5	-0.38855	-1.0806	0.35196	0.91185
GO:0140678	Molecular function inhibitor activity	8	0.4274	1.2934	0.18129	0.91332
GO:0016874	Ligase activity	6	-0.21193	-0.6335	0.9142	0.91655
GO:0016829	Lyase activity	7	0.39201	1.1344	0.30317	0.91726
GO:0022804	Active transmembrane transporter activity	14	0.31039	1.182	0.23784	0.91856
GO:0140297	DNA-binding transcription factor binding	11	0.23722	0.83907	0.67507	0.92127
GO:0003729	Mrna binding	5	0.22858	0.58314	0.94836	0.9408
GO:0001216	DNA-binding transcription activator activity	7	0.29024	0.85928	0.64231	0.9479
GO:0003924	Gtpase activity	5	-0.25338	-0.7095	0.84807	0.95091
GO:0019001	Guanyl nucleotide binding	6	-0.25424	-0.7549	0.793	0.95665
GO:0004518	Nuclease activity	8	0.19718	0.60539	0.92342	0.97264

ES: Enrichment Score; NES: Normalized Enrichment Score; FDR: Significance level

Table 4S. KEGG pathways HCC1937 cells*

Pathway	ID	Input	Total	P-Value	Corrected P-Value
**Down-expressed genes**					
Herpes simplex virus 1 infection	hsa05168	56	492	1.60e-11	4.65e-09
Homologous recombination	hsa03440	13	41	7.17e-08	1.04e-05
Proteoglycans in cancer	hsa05205	26	203	5.74e-07	5.56e-05
Pathways in cancer	hsa05200	45	530	3.39e-06	2.47e-04
Cellular senescence	hsa04218	21	160	4.81e-06	2.68e-04
Ras signaling pathway	hsa04014	25	232	1.48e-05	6.17e-04
Small cell lung cancer	hsa05222	14	93	4.73e-05	1.72e-03
NOD-like receptor signaling pathway	hsa04621	20	178	6.04e-05	1.95e-03
Regulation of actin cytoskeleton	hsa04810	22	214	8.88e-05	2.27e-03

MAPK signaling pathway	hsa04010	27	295	9.36e-05	2.27e-03
Epstein-Barr virus infection	hsa05169	20	201	2.69e-04	6.03e-03
PI3K-Akt signaling pathway	hsa04151	29	354	3.04e-04	6.31e-03
Hepatitis B	hsa05161	17	163	4.62e-04	8.30e-03
Thyroid hormone signaling pathway	hsa04919	14	119	4.85e-04	8.30e-03
Axon guidance	hsa04360	18	181	5.36e-04	8.35e-03
Focal adhesion	hsa04510	19	199	6.03e-04	8.35e-03
Human cytomegalovirus infection	hsa05163	20	225	9.97e-04	1.21e-02
**Overexpressed genes**					
Pathways in cancer	hsa05200	18	530	7.73e-08	1.46e-05
Transcriptional misregulation in cancer	hsa05202	11	186	1.64e-07	1.46e-05
MAPK signaling pathway	hsa04010	12	295	2.02e-06	1.24e-04
Metabolic pathways	hsa01100	26	1433	1.39e-05	5.59e-04

*P-Value <0.001, input >10

Table S5. KEGG pathways MDA-MB-231 cells*

Pathway	ID	Input*	Total	P-Value	Corrected P-Value
**Down-expressed genes**					
Herpes simplex virus 1 infection	hsa05168	43	492	5.14e-09	1.35e-06
Proteoglycans in cancer	hsa05205	23	203	3.33e-07	4.37e-05
Focal adhesion	hsa04510	22	199	8.68e-07	7.58e-05
Protein processing in endoplasmic reticulum	hsa04141	19	165	2.76e-06	1.81e-04
Small cell lung cancer	hsa05222	14	93	3.58e-06	1.88e-04
ECM-receptor interaction	hsa04512	13	86	7.61e-06	2.80e-04
Regulation of actin cytoskeleton	hsa04810	21	214	8.38e-06	2.80e-04
Pathways in cancer	hsa05200	37	530	8.56e-06	2.80e-04
MAPK signaling pathway	hsa04010	25	295	1.29e-05	3.64e-04
Estrogen signaling pathway	hsa04915	16	138	1.53e-05	3.64e-04
PI3K-Akt signaling pathway	hsa04151	27	354	3.37e-05	7.36e-04
Toxoplasmosis	hsa05145	13	113	1.01e-04	2.03e-03
mTOR signaling pathway	hsa04150	15	153	1.58e-04	2.96e-03
Hypertrophic cardiomyopathy (HCM)	hsa05410	11	90	2.10e-04	3.51e-03
Signaling pathways regulating pluripotency of stem cells	hsa04550	14	140	2.14e-04	3.51e-03
Hepatitis B	hsa05161	15	163	2.97e-04	4.33e-03
Dilated cardiomyopathy (DCM)	hsa05414	11	96	3.48e-04	4.80e-03
Transcriptional misregulation in cancer	hsa05202	16	186	3.81e-04	5.00e-03
Human papillomavirus infection	hsa05165	23	330	4.16e-04	5.20e-03
**Overexpressed genes**					
Metabolic pathways	Hsa01100	24	1433	2.49e-04	2.14e-02

*P-Value <0.001, input >10
